# Inflammatory Mediators in Atherosclerotic Vascular Remodeling

**DOI:** 10.3389/fcvm.2022.868934

**Published:** 2022-05-04

**Authors:** Bryce R. Evans, Anaïs Yerly, Emiel P. C. van der Vorst, Iris Baumgartner, Sarah Maike Bernhard, Marc Schindewolf, Yvonne Döring

**Affiliations:** ^1^Division of Angiology, Swiss Cardiovascular Center, Inselspital, Bern University Hospital, University of Bern, Bern, Switzerland; ^2^Department for BioMedical Research (DBMR), University of Bern, Bern, Switzerland; ^3^Institute for Cardiovascular Prevention (IPEK), Ludwig-Maximilians-University Munich (LMU), Munich, Germany; ^4^DZHK (German Centre for Cardiovascular Research), Partner Site Munich Heart Alliance, Munich, Germany; ^5^Institute for Molecular Cardiovascular Research (IMCAR) and Interdisciplinary Center for Clinical Research (IZKF), RWTH Aachen University, Aachen, Germany; ^6^Department of Pathology, Cardiovascular Research Institute Maastricht (CARIM), Maastricht University Medical Centre, Maastricht, Netherlands

**Keywords:** atherosclerosis, remodeling, inflammatory mediator, chemokines, cytokines

## Abstract

Atherosclerotic vascular disease remains the most common cause of ischemia, myocardial infarction, and stroke. Vascular function is determined by structural and functional properties of the arterial vessel wall, which consists of three layers, namely the adventitia, media, and intima. Key cells in shaping the vascular wall architecture and warranting proper vessel function are vascular smooth muscle cells in the arterial media and endothelial cells lining the intima. Pathological alterations of this vessel wall architecture called vascular remodeling can lead to insufficient vascular function and subsequent ischemia and organ damage. One major pathomechanism driving this detrimental vascular remodeling is atherosclerosis, which is initiated by endothelial dysfunction allowing the accumulation of intimal lipids and leukocytes. Inflammatory mediators such as cytokines, chemokines, and modified lipids further drive vascular remodeling ultimately leading to thrombus formation and/or vessel occlusion which can cause major cardiovascular events. Although it is clear that vascular wall remodeling is an elementary mechanism of atherosclerotic vascular disease, the diverse underlying pathomechanisms and its consequences are still insufficiently understood.

## Introduction

Inflammatory mediators such as chemokines and cytokines are quickly released by a multitude of cell types during inflammation and trauma and thereby orchestrate a vital immune response and vascular remodeling. These interactions can occur in either an autocrine or paracrine fashion resulting in structural and functional changes of the vascular wall. However, during chronic inflammatory conditions these mediators can also cause tissue damage or (irreversible) tissue remodeling, a term referring to structural and functional changes of the arterial vessel wall (1). A chronic disease that is characterized by such arterial vessel remodeling is atherosclerotic vascular disease, which is the most common cause of cardiovascular disease (CVD) (2). Mechanisms involved in atherosclerotic arterial remodeling include hyperplasia of the arterial intima and media, changes in vascular collagen and elastin, endothelial dysfunction, and arterial calcification. Identifying the cross talk between cells of the vasculature which maintain or disrupt vascular homeostasis and result in vascular remodeling may offer strategic insight for CVD prevention.

Atherosclerosis is initiated by endothelial dysfunction allowing lipids to accumulate in the intima, which subsequently results in intimal inflammation driving (persistent) vascular changes (3). Various inflammatory mediators comprising of modified lipids, such as *oxidized low-density lipoprotein* (oxLDL), but also chemokines, cytokines and lipid mediators further foster the endothelial dysfunction and increase vascular permeability. This increased permeability further stimulates the influx and accumulation of lipids and immune cells in the intimal layer of the vascular wall, resulting in a vicious circle (3). The immune cell infiltration into the vessel wall is further increased by the upregulation of adhesion molecules on the endothelium, stimulated by the inflammatory environment. This further induces the arrest of monocytes and other leukocytes onto the vessel wall which subsequently transmigrate into the intima (4, 5). Infiltrated monocytes subsequently differentiate into macrophages which will engulf excess lipids and develop into lipid laden foam cells. Due to the excess uptake of lipids, these foam cells will eventually undergo apoptosis and necrosis, resulting in the formation of a necrotic core in atherosclerotic lesions (6). Accumulation of such necrotic debris leads to the continued release of toxic and pro-inflammatory stimuli in the intima, further promoting inflammation, remodeling and vulnerability of the plaque.

Key cells in shaping the vascular architecture are *vascular smooth muscle cells* (VSMCs), which are normally present in the arterial media and express a range of “SMC markers” including *smooth muscle cell myosin heavy chain* (MYH11), *smooth muscle cell actin* (SM-α), smoothelin and others. These VSMCs are considered to have a contractile phenotype, which is important to maintain the vascular tone. However, during atherosclerosis formation a phenotype switch is induced by inflammatory mediators resulting in the transition from a contractile phenotype to a synthetic phenotype. Additionally, VSMCs will proliferate and migrate from the media into the intima where they will produce *extracellular matrix* (ECM) to form a fibrous cap and stabilize the atherosclerotic lesions. Moreover, VSMCs with a synthetic phenotype adopt macrophage-like characteristics and can also develop into SMC-derived foam cells (2). Additionally and especially in later stages of lesion development, synthetic VSMCs also produce and secrete matrix metalloproteinases (MMPs) resulting in greater proteolytic activity toward elastin and collagen, which destabilizes the plaque and increases the risk of plaque rupture and thrombus formation (7).

This review aims to draw attention to the main inflammatory mediators involved in vascular remodeling seen in atherosclerosis.

## Chemokines

Chemokines are a family of chemoattractant cytokines secreted by various cells, which play a vital role in cell migration from the bloodstream into tissues. They induce cell movement in response to and toward a chemokine gradient also referred to as chemotaxis (8). In addition, chemokines play an important role in various cellular functions including proliferation, survival and differentiation (9). Chemokines can be classified into four structural subfamilies, CC, CXC, CX_3_C and C based on the location of the key cysteine residues in the disulfide bonding which are either juxtaposed (CC) or separated by 1 or 3 amino acids (CXC and CX_3_C) respectively (10). Chemokines initiate cellular responses through interaction with seven-transmembrane G-protein coupled receptors (GPCRs), more specifically classical chemokine receptors, or with atypical chemokine receptors (ACKRs), which do not signal through G-proteins (11).

CC chemokines have at least 27 distinct members reported in mammals, called CC chemokine ligands (CCL) and are typically responsible for the induction of leukocyte migration (10, 11). Currently, 17 different CXC chemokines have been described in mammals, which can be further subdivided into two subcategories, based on the presence or absence a specific amino acid sequence of glutamic acid-leucine-arginine immediately before the first cysteine of the CXC motif (12). The subgroup of CXC chemokines with this sequence specifically induce the migration of neutrophils, while the subgroup without this sequence typically attract lymphocytes. More unique is CX_3_CL1, which possesses three amino acids between the two cysteines and is also termed CX_3_C chemokine or fractalkine (12) and the two C chemokines XCL1 (lymphotactin-α) and XCL2 (lymphotactin-β) (12).

The remainder of this chapter will focus on individual chemokines that have been shown to be involved in vascular remodeling during inflammation with a particular focus on atherosclerosis.

### CCL2

CCL2 (Table 1) also known as monocyte chemoattractant protein-1 is a member of the CC chemokine subfamily and exhibits potent chemotactic activity toward monocytes and T lymphocytes (36). Jones et al. (37) demonstrated that activated VSMCs isolated from mice secreted more CCL2 and CXCL1 and *in vivo* knockout of *Protease-Activated Receptor* 2 (PAR2) in vascular cells reduced their expression of CCL2/CXCL1 and resulted in a reduction of macrophage content in atherosclerotic lesions compared to the control animals. Additional findings demonstrated increased plaques stability, increased smooth *muscle actin alpha* 2 ACTA-2, collagen content and reduced interleukin-1 (IL)-1 and tumor necrosis factor (TNF)-α. This suggests that CCL2 acts on macrophage chemotaxis into the lesion in a paracrine fashion (**Figure 2**). Furthermore, CCL2 may interact on VSMC via an autocrine mechanism to stimulate the phenotypic changes toward a synthetic phenotype. Besides promoting the transmigration of circulating monocytes, CCL2 also promotes cytokine production and adhesion molecule expression on monocytes (38). CCL2 expression is induced by inflammatory cytokines, growth factors, or complement factors in monocytes, ECs, and VSMCs (39, 40). Furthermore, CCL2 seems to be an important chemokine in the development of atherosclerosis, since its expression has been detected in atherosclerotic lesions but not in vessels obtained from healthy individuals (38, 41) and in patients with MI (36, 42). Enhanced CCL2 within the lesion is correlated with histopathologic, molecular, and clinical hallmarks of plaque vulnerability, clearly suggesting that this chemokine also plays a role in advanced stages of lesion development (43). Interestingly, circulating CCL2 levels also correlate with subclinical atherosclerosis disease severity in postmenopausal women and may act as a potential early biomarker in this population (44). Further studies have demonstrated that circulating myeloid cells deposit CCL2 on the arterial endothelium to enhance monocyte recruitment and thereby drive atherogenesis (15).

**Table 1:** Overview of chemokines involved in atherosclerosis remodeling and their physiological effect.

**Chemokines**	**Receptors**	**Cells affected**	**Proposed effect in vascular remodeling**	**References**
CCL2	CCR2	Monocytes	Recruitment	(13)
				(14)
				(15)
		VSMCs	Migration via PI3Kγ signaling	(16)
			Activation of NF-kβ and AP-1 leading to cytokine secretion	(17)
			Proliferation	
CCL5 (RANTES)	CCR1	Monocytes	Recruitment, Arrest, infiltration	(18)
	CCR3	Macrophages	Differentiation into foam cells	(19)
	CCR5	VSMCs	Proliferation	(19)
			Phenotypic switch from contractile to synthetic repair cell	
CCL19/CCL21	CCR7	Monocytes	Recruitment	(20)
		Macrophages	Foam cell formation	(21)
				(22)
		VSMCs	Proliferation	(22)
			Increase MMP-1 expression	
CXCL10	CXCR3	CD4+ T lymphocytes	Recrutment of CD4+ T lymphocytes and Tregs	(23)
		Endothelial cells	Reduced wound healing	(24)
CXCL12	CXCR4	Monocytes	Recruitment	(25)
		Macrophages	Differentiation into foam cell	(25)
		VSMCs	Migration	(26)
			Secretion of collagen	(27)
				(28)
CXCL16	CXCR6	Platelets	Deposition on ECs	(29)
		Macrophages	Differentiation into foam cells	(30)
				(31)
		VSMCs	Differentiation into foam cells	(30)
				(31)
CX3CL1 (Fractalkine)	CX3CR1	Monocytes	Recruitment and adhesion	(32)
				(33)
		VSMCs	Proliferation	(34)
				(35)

CCL2 is also involved in vascular remodeling as it has been shown to stimulate the binding activity of *nuclear factor kappa-light-chain-enhancer of activated B cells* (NFκB) to *activator protein-*1 (AP-1) in cultured human VSMCs (HVSMCs) grown from unused portions of saphenous veins harvested during coronary artery bypass surgery (17). In addition, CCL2 was reported to induce the proliferation and IL-6 secretion from HVSMCs *in vitro* (17). Furthermore, recombinant CCL2 stimulated HVSMC proliferation *in vitro via* AP-1, which was inhibited by *mitogen-activated protein kinase* (MEK)-1 and MEK2 inhibitor treatment (17). This suggests that CCL2 induces differential activation of NFκB and AP-1 leading to cytokine secretion and proliferation in human VSMCs. Moreover, pharmacological *phosphatidylinositol* 3*-kinase gamma* (PI3Kγ) inhibition, inactivation of PI3Kγ, as well as genetic deletion of PI3Kγ in mice was used to study the role of CCL2 on the *platelet-derived growth factor* (PDGF) signaling pathway and migration processes in primary aortic VSMCs (16). A wound healing assay illustrated that CCL2 is crucial for VSMC migration via PI3Kγ signaling as blocking the CCL2/CCR2 pathway or the inhibition of PI3Kγ reduced PDGF-stimulated aortic VSMC migration by 50% (16). Furthermore, a *low-density lipoprotein receptor* knock-out (*Ldlr*^−/−^) mouse model fed a *Western diet* (WD) for 8-weeks treated with a PI3Kγ inhibitor showed decreased atherosclerotic lesion size and increased plaque collagen content (45). Finally, VSMCs isolated from PI3Kγ-deficient mice (*PI*3*K*γ^−/−^), or mice expressing an inactive PI3Kγ, termed PI3Kγ^KD/KD^, showed reduced migration when compared to the control cells in response to CCL2 and PDGF (16). Combined these results demonstrate that CCL2 plays a role in lesion formation by promoting VSMC migration in a PI3Kγ dependent manner but can also result in decreased plaque stability by reducing the collagen formation.

Another study demonstrated that a CCL2 competitor (PA508) reduced inflammatory monocyte recruitment, limited neointimal hyperplasia, and attenuated myocardial ischemia/reperfusion injury in an *Apolipoprotein E* knock-out (*Apoe*^−/−^) mouse model, highlighting the potential of PA508 as novel therapeutic approach to treat MI (13). However, the study did not examine mice on a WD to investigate the therapeutic potential of PA508 on atherosclerosis development. Nevertheless, another study treating *Apoe*^−/−^ mice, fed with a WD for 6-weeks, daily with a CCR2 antagonist (INCB3344) revealed reduced circulating CCR2^+^ monocytes and diminished atherosclerotic plaques in both the carotid artery and the aortic root (14), proving the therapeutic potential of CCL2/CCR2 targeting for atherogenesis.

**Figure 1:**
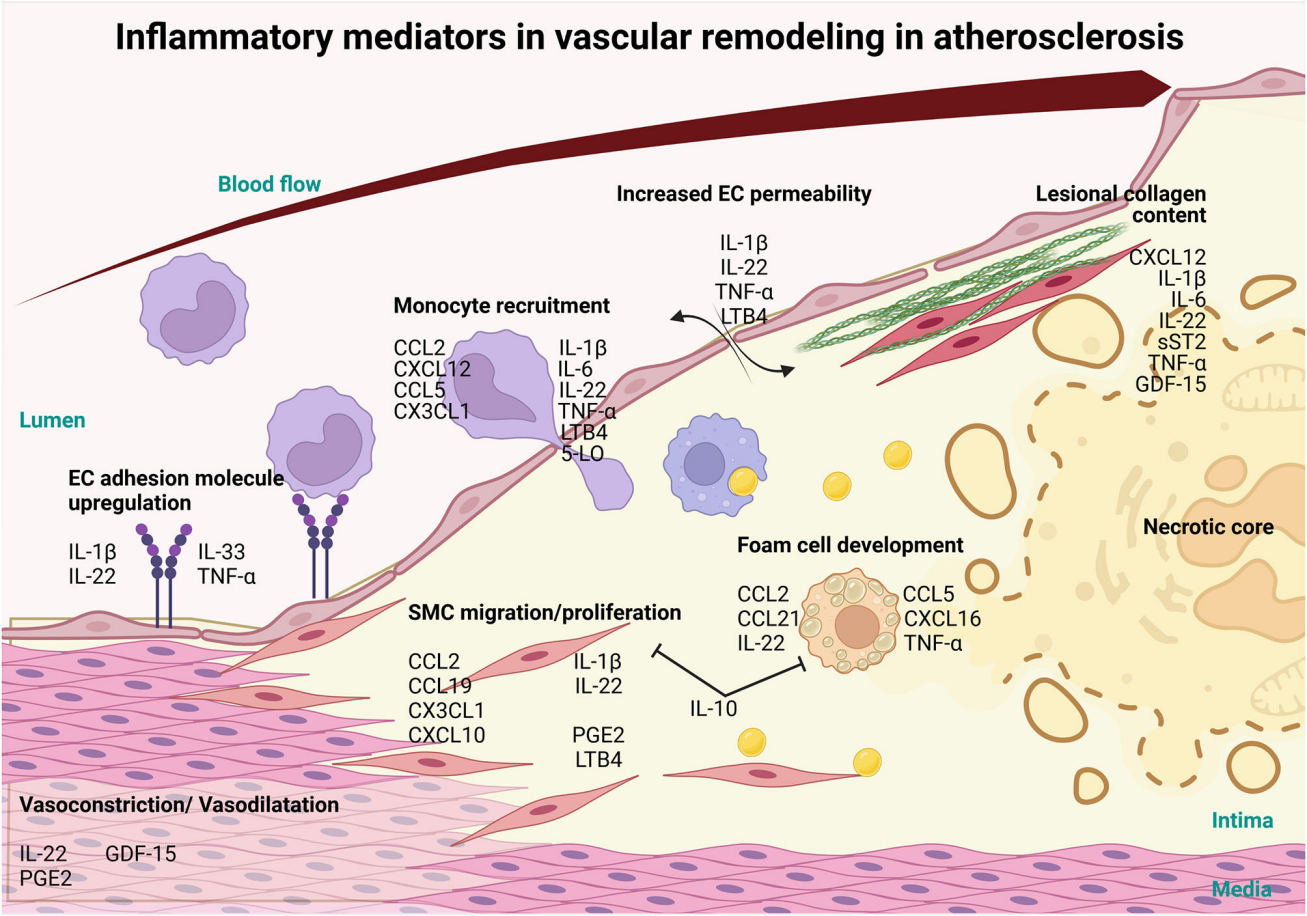
Involvement of inflammatory mediators in vascular remodeling in atherosclerosis. Inflammatory mediators, such as interlukin-1β (IL-1β), IL-22, IL-33 and tumor necrosis factor-α (TNFα) cytokines can influence the progression of atherosclerosis and CVD *via* the activation of the endothelium resulting in the upregulation of adhesion molecules. Furthermore, these mediators increase vascular permeability, through IL-1β, IL-22, TNFα and LTB4 and along with the adhesion molecule upregulation allows for the infiltration of monocytes and other immune cells recruited *via* chemokine ligand 2 (CCL2), C-X-C Motif Chemokine Ligand 12 (CXCL12), CCL5, CX3CL1, IL-1β, IL-6, IL-22, TNF-α Leukotriene B4 (LTB4), 5-LO. Mediators like CCL2, CCL19, CXCL10, CX3CL1, IL-1β, IL-22, Prostaglandin E_2_ (PGE2) and LTB4 also induce the migration and proliferation of VSMCs into the intima and affect the production of collagen, which, in turn, modulates plaque stability. Foam cell formation is initiated by CCL2, CCL5, CCL21, CXCL16, IL-22 and TNFα and exhausted foam cells undergoing apoptosis and necrosis to establish the necrotic core of the lesion. On the other hand, IL-10, a potent anti-inflammatory cytokine, prevents the formation of foam cells and SMC migration and proliferation. As the lesion grows, blood vessel lumen is narrowing eventually causing vessel occlusion which may lead to major adverse cardiovascular complications. Inflammatory mediators CXCL12, IL-1β, IL-6, IL-22, soluble suppression of tumorigenesis-2 (sST2), TNFα and Growth/Differentiation Factor-15 (GDF-15) also play a role in the stability of the lesion by controlling collagen in the fibrous cap. In addition, mediators like LL-22, GDF-15 and PGE2 regulate vasoconstriction and vasodilation of the arteries thereby controlling blood pressure and ensuring proper vascular function (this figure was made with Biorender.com).

Overall, as CCL2 has been shown to be overexpressed in atherosclerotic lesions it is likely that it plays a prominent role in vascular remodeling of VSMCs in atherosclerosis (Figure 1). CCL2 may stabilize the plaque by fostering migration of medial VSMCs into the intima and increase their proliferation while in parallel, recruitment of arterial leukocytes into the lesion triggers atherogenesis (15). Furthermore, therapeutic strategies, such as the PI3Kγ inhibition or CCL2/CCR2 pathway inhibition reveal a decrease in lesion size in mice due to the reduced VSMC migration (45) (Table 2). Nevertheless, further research is necessary to demonstrate whether the putative beneficial effects of CCL2 on VSMCs, e.g., increased proliferation and subsequent increase of plaque stability, outbalance its pro-atherogenic effects on leukocyte recruitment.

**Table 2:** Targeting chemokines as therapeutic treatments in vascular remodeling and CVD.

**Chemokine**	**Therapeutic Treatment**	**Clinical trials**	**Animal experimenta-tion**	**Outcomes on atherosclerosis**	**References**
CCL2	Pharmacological phosphatidylinositol 3-kinase gamma (PI3Kγ) inhibitor	–	✓	Reduces PDGF-Stimulates aortic VSMC migration by 50%	(16)
	CCL2 competitor (PA508)	–	✓	Reduces inflammatory monocyte recruitment	(13)
				Limited neointimal hyperplasia and attenuates myocardial ischemia/reperfusion injury	
	CCR2 antagonist (INCB3344)	–	✓	Reduces circulating CCR2+ monocytes, Diminished atherosclerotic plaques	(14)
CCL5	CCL5 antagonist (Met-RANTES)	–	✓	Reduced atherosclerotic lesion size	(46)
				Reduction in foam cells	
	MKEY	–	✓	Decreases leukocyte recruitment into infarcted tissue Decreases release of NETs	(47)
CCL19/CCL21	anti-CCL21 monoclonal antibody	–	✓	Reduction of the infarction size after AMI	(20)
CXCL10	pharmaceutical antagonist specific for CXCR3 (NBI-74330)		✓	Reduced lesion size, CD4+ T lymphocytes content and increased Tregs content	(23)
CXCL12	–	–	–	–	–
CXCL16	–	–	–	–	–
CX3CL1	CX3CL1-Fc fusion protein	–	✓	Reduces atherosclerotic lesions size, independent of the diet	(33)

### CCL5

CCL5, a chemokine which is also known as RANTES, can bind to a plethora of receptors including CCR1, CCR3 and CCR5 (Table 1) (48). Platelet-derived deposition of CCL5 on the activated endothelium results in monocyte arrest (49), which appears to be dependent on P-selectin (50). Wire-injury induced neointima formation in the carotid artery of *Apoe*^−/−^ mice showed that the systemic inhibition of CCL5 or P-selectin deficiency hindered neointima formation as well as monocyte infiltration (50). Furthermore, it could be demonstrated that *CCL*5 mRNA and protein levels were upregulated in the aortic intima of *Ldlr*^−/−^ mice fed a WD for 3 weeks and a function-blocking antibody to CCR5 significantly reduced monocyte recruitment into the lesions (18). Further investigations using a *bone marrow transplantation* (BMT) experiment demonstrated that hematopoietic CCL5 regulates monocyte recruitment and accumulation of macrophages in the lesions after 3 weeks of cholesterol-rich diet feeding of *Ldlr*^−/−^ mice. However, after 6 weeks of cholesterol-rich diet, CCL5 only plays a minor role in the recruitment of monocytes into the lesions suggesting that CCL5 only plays a role in early stages of lesion formation and subsequent vascular remodeling (18).

**Figure 2:**
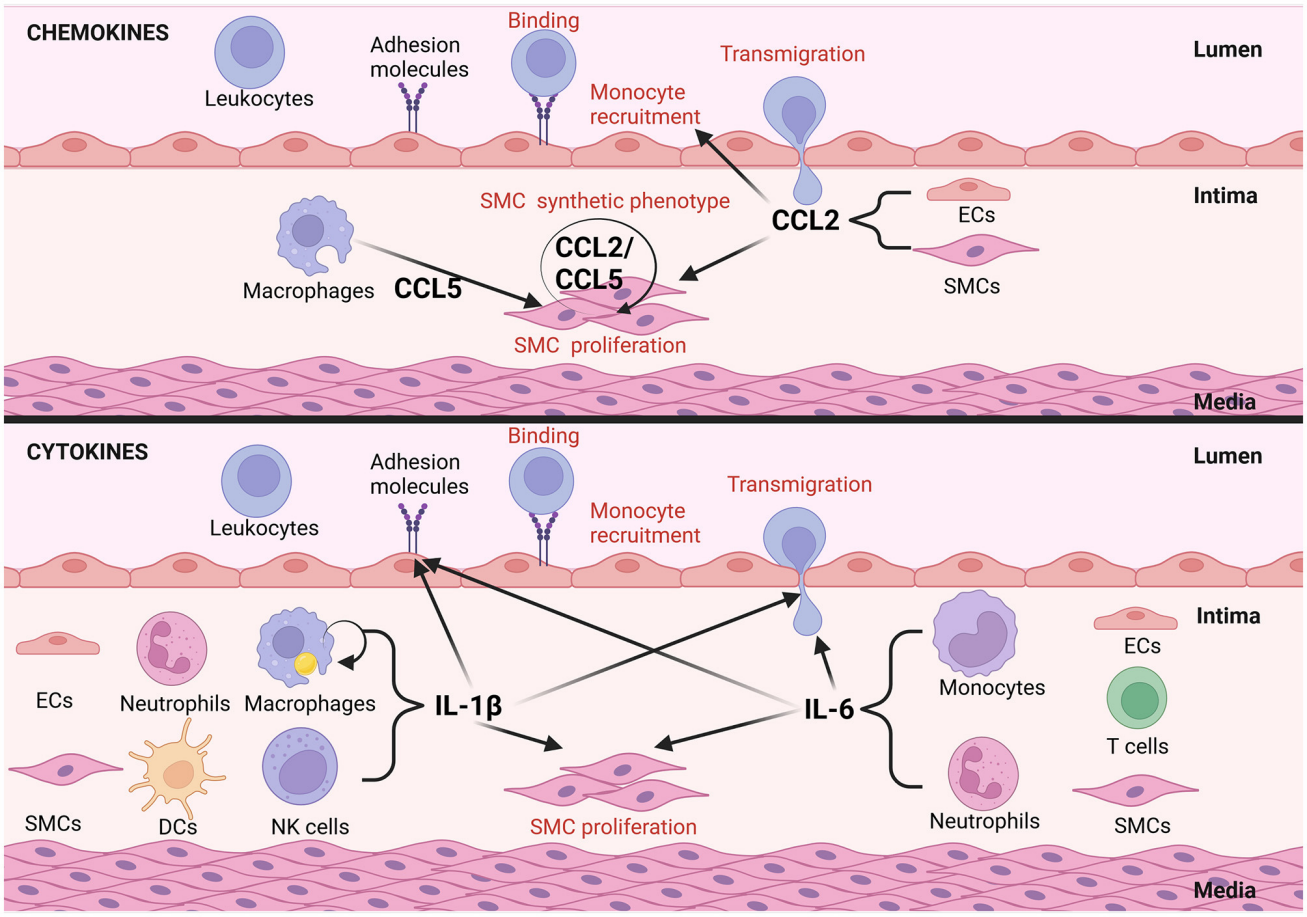
Examples of autocrine/paracrine interactions of inflammatory mediators in vascular remodeling in atherosclerosis. Inflammatory mediators can act in an autocrine manor, CCL2 and CCL5 for example activate VSMCs to undergo phenotypical changes in atherosclerosis. EC and VSMC derived CCL2 can further foster cross-talk between them promoting remodeling. CCL2 from ECs triggers synthetic differentiation of VSMCs and CCL2 from VSMCs promotes monocyte recruitment. Alternatively, mediators like interlukin-1β, expressed by dendritic cells (DCs) and natural killer (NKs) cells promote the upregulation of adhesion molecule expression by ECs, transmigration of circulating leukocytes and VSMC proliferation in a paracrine fashion. IL-1β in addition can act upon macrophages to promote IL-1β secretion in an autocrine manner. IL-6 secreted by ECs, monocytes, T cells, neutrophils and VSMCs promotes adhesion molecule expression by ECs as well as transmigration of circulating immune cells into the atherosclerotic plaque (this figure was made with Biorender.com).

Similar results were seen in a 2-week WD *Ldlr*^−/−^ mouse model where mice were treated with a CCL5 antagonist (Met-RANTES). Treated animals had reduced atherosclerotic lesion size and a reduction in relative lesional foam cell content compared to the control group (46). Thus, CCL5 mediates vascular remodeling in atherogenesis *via* monocyte arrest and infiltration into the lesion and facilitates the differentiation and development of monocytes into foam cells, although the exact underlying mechanisms behind this CCL5-induced foam cell formation remain to be elucidated. Another study, using *CCL*5^−/−^*CCR*5^–/−^ mice on a 12-week WD, showed significantly reduced expression of the synthetic markers osteopontin and *proliferating cell nuclear antigen* (*PCNA*), while the expression of the contractile VSMC marker SMα was increased in the thoracoabdominal aorta compared to the control group (19). Furthermore, *in vitro* culturing of human aortic SMCs (HASMCs) with palmitic acid resulted in an increased expression of proliferative and synthetic phenotype markers while inhibition of CCR5 using the antagonist maraviroc or RNA interference prevented HASMC proliferation and synthetic phenotype formation (19). Macrophages from stroke patients, exhibit an increased expression of CCL5 which signals through CCR5 on VSMCs driving their proliferation and dedifferentiation, suggesting a paracrine relationship between these macrophages and vascular VSMCs [(19, 51), p. 153]. These data suggest that CCL5 induces VSMC proliferation and phenotypic switching from a contractile to synthetic phenotype via CCR5 (Figure 2). The latter would argue for a pro-atherosclerotic role of CCL5 and induction of a synthetic VSMC phenotype promoting vascular remodeling. However, Lin et al. did not backcross the mice on an atherosclerotic background such as *Apoe*^−/−^ or *Ldlr*^−/−^, making it impossible to determine whether CCL5 also promotes a synthetic VSMC phenotype during atherogenesis.

CCL5 can also heterodimerize with CXCL4, another platelet-derived chemokine deposited onto vascular endothelium (52). Enhanced CXCL4 presence in human plasma correlates with the severity of human atherosclerotic disease and the platelet specific deletion of CXCL4 decreases atherosclerotic lesions in mice (53, 54). Furthermore, interfering with this heterodimer via the use of the cyclic peptide MKEY resulted in decreased leukocyte recruitment and release of NETs (47). All in all, these studies suggest that CCL5 at least exerts part of its atherogenic effects by heterodimerizing with CXCL4.

CD146, a cell adhesion molecule expressed on arterial endothelium was also implicated in CCL5-mediated changes to the vascular wall. *CD*146^−/−^*Apoe*^−/−^ mice fed a WD for 24 weeks displayed lesions with a greater neutrophil and macrophage content correlating with the upregulation of CCL5 secretion when compared to the control group (55). Furthermore, neutrophil recruitment was increased in CD146-deficient mice 12 h after thioglycolate-induced peritonitis, whereby this increased recruitment correlated with the enhanced CCL5 secretion in the peritoneal cavity. The same study showed that mice treated with maraviroc between week 12 to 24 of WD feeding showed smaller atherosclerotic lesions and reduced neutrophilia in *CD*146^−/−^*Apoe*^−/−^ mice to the same level found in *Apoe*^−/−^ mice (55). These data suggest that CCL5 plays a prominent role in neutrophil recruitment and foam cell formation in atherosclerotic lesions and identifies CD146 agonists as potential therapeutic targets to lower CCL5 levels (Figure 1). Hence, increased levels of CCL5 may act as a biomarker for the severity of atherosclerosis and may even be a potent therapeutic target for SMC proliferation and phenotype switching (Table 2).

### CCL19/CCL21

Chemokines CCL19 and CCL21 and their receptor CCR7 (Table 1) are associated with the modulation of inflammatory responses in lymphoid and nonlymphoid tissues, including atherosclerotic lesions (56–58). Elevated levels of circulating CCL19 and CCL21 can be found in patients with unstable angina pectoris compared to controls (57) and enhanced levels of these chemokines were also observed in patients with carotid atherosclerosis, both systemically (CCL21) and within the lesion (CCL19 and CCL21) (22).

In a mouse model studying MI, the inhibition of CCL21 via intravenous injection of anti-CCL21 monoclonal antibodies led to the reduction of the infarction size after acute MI (20). Anti-CCL21 monoclonal antibody treatment further resulting in reduced MMP-9 and total collagen content in the myocardium. CCL21 was also shown to increase the binding of *acetylated LDL* (ac-LDL) to macrophages, inducing the up-regulation of *LDL receptor-*1 (LOX-1), a scavenger receptor of ox-LDL which has been shown to drive foam cell formation (21, 22). Furthermore, the lipid droplet marker *adipose differentiation-related protein* (ADRP) was upregulated in CCL21-treated macrophages indicating that CCL21 induces foam cell formation (59).

CCL19 has been shown to induce proliferation of VSMCs and results in increased MMP-1 serum levels (22), which positively correlates with total plaque burden (60). Using a BMT experiment of either *CCL*19^−/−^ or *CCL*21^−/−^ bone marrow into *Ldlr*^−/−^ mice it was further demonstrated that CCL21 directs leukocyte homing into atherosclerotic lesions, whereas CCL19 induces the activation of leukocytes, lipid uptake by macrophages and foam-cell formation (61). Thus, CCL19 is involved in vascular remodeling in atherosclerosis, by inducing VSMC proliferation and enhancing protein expression of MMP-1 in VSMCs, which acts to destabilize the plaques, while both CCL19 and CCL21 foster macrophage foam cell formation (Figure 1). Blocking CCL19 and/or CCL21 may therefore be a potent therapeutic target to decrease the lipid content in lesions and increase plaque stability (Table 2).

### CXCL10

EC, VSMCs, and macrophages all express CXCL10 during atherosclerosis while its receptor CXCR3 is mainly expressed on CD4^+^ T cells (Table 1) (62). Evidence from *Apoe*^−/−^ *Cxcl*10^−/−^ mice on a WD for 6 and 12 weeks demonstrated attenuated lesions, reduced CD4^+^ T cell content and increased regulatory T cells (Tregs) markers within the lesions, suggesting an increase in Tregs within the lesion of *Apoe*^−/−^ *Cxcl*10^−/−^ compared to the control (63). Furthermore, *Ldlr*^−/−^ mice fed a 2-week WD before a collar placement and a further 8 week WD with daily treatments of a pharmaceutical antagonist specific for CXCR3 (NBI-74330) demonstrated a reduced lesion size in the aorta and aortic root compared to the control (23). Further investigation showed reduced CD4^+^ T cells in the lesion and greater expression of genes associated with greater presence of Tregs in the plaques from mice treated with NBI-74330. This suggests that CXCL10 plays a prominent role in vascular remodeling and may be a potential therapeutic target (Table 2). Further experiments were conducted in *Apoe*^−/−^ mice fed a WD for 2 weeks followed by induction of an unstable plaque with a flow-altering device around the carotid artery. These animals were further fed a WD for 9 weeks and treated with a bioactivity-neutralizing monoclonal CXCL10 antibody (MAB466) (64). The CXCL10 antibody treatment resulted in a more stable lesion phenotype with increased VSMCs content compared to untreated controls. Hence, CXCL10 may also influence VSMC behavior affecting plaque stability, potentially making CXCL10 an attractive therapeutic target for vascular remodeling (65). Adding to this, in PI3KγKD/KD mice treated with the CXCL10 antibody (MAB466) demonstrated that CXCL10 production by VSMCs inhibited endothelial healing in a wire induced scratch model (24). This suggests that CXCL10 also has a paracrine relationship with endothelial healing and may play a role in maintaining endothelial dysfunction in atherosclerosis. However, this would need to be further investigated in an atherosclerotic mouse model on a WD. Furthermore, studies using an *in vitro* model in which human endothelial cells (SGHEC-7) were co-cultured with the human VSMC cell line SGHVSMC-9 demonstrated that CXCL10 expression contributes to remodeling by altering the motility and differentiation of the VSMCs (66). Future studies could investigate the role of CXCL10 on the phenotypic switching of VSMCs within atherosclerosis.

### CXCL12

The chemokine CXCL12 (Table 1) is highly expressed in human atherosclerotic lesions (32, 67) and genome-wide association studies noted that the genomic locus 10q11, hosting the *CXCL*12 gene and the intergenic single nucleotide polymorphism (SNP) rs2802492 located near *CXCL*12, are independently associated with CXCL12 plasma levels and *coronary artery disease* (CAD) risk (68, 69). Furthermore, a causative detrimental role of enhanced CXCL12 titers in CAD was described (70) and may also account for CVD in general, rendering CXCL12 a useful biomarker for CVD risk. In line with this, mouse studies could show that EC-specific *CXCL*12 knock out in *Apoe*^−/−^mice fed a WD for 12 weeks reduced the lesion area in the thoracoabdominal aorta and the aortic arch as well as CXCL12 plasma levels compared to control mice (69). Furthermore, a positive correlation between CXCL12 plasma levels and lesion area was seen in the control but not in EC-CXCL12 deficient mice. Combined, these observations suggest that EC-derived CXCL12 promotes atherosclerosis and vascular remodeling (69).

*In vitro* assays exploring the effects of ox-LDL treatment demonstrated an increase in CXCL12 protein and mRNA expression compared to untreated controls for both THP-1 cells (human monocyte cell line derived from a patient with acute monocytic leukemia) and HASMCs (71). Furthermore, Gao et al. (25) showed that CXCL12-treated THP-1 cells expressed less *ATP Binding Cassette Subfamily A Member* 1 (*ABCA*1) and subsequently resulted in a reduced cholesterol efflux capacity toward apolipoprotein A1 (ApoA-I). The same study also revealed that systemic overexpression of *CXCL*12 in *Apoe*^−/−^ mice, using a lentivirus, resulted in increased lesion formation as well as macrophage accumulation in the plaques. A more detailed analysis demonstrated decreased *ABCA*1 mRNA expression in lesional macrophages in *Apoe*^−/−^ mice which overexpressed CXCL12 supporting the observations made *in vitro* (25). Together, these data suggest that CXCL12 promotes the formation of macrophage-derived foam cells in atherosclerotic lesions via inhibition of *ABCA*1 expression. The potential of CXCL12 to promote foam cell formation by VSMCs or any other analysis of VSMCs was not investigated in the *in vivo* model in this study and should therefore be a subject for future research.

In contrast to the above described observations, CXCL12 is upregulated in the artery via lysophosphatidic acid in response to vessel injury, and the increase in circulating CXCL12 induces the migration of a subtype of Sca-1^+^ Lin^−^ *smooth muscle progenitor cells* (SPCs) (26, 27). Intravenous injection of CXCL12 in *Apoe*^−/−^ mice fed a WD resulted in increased plaque stabilization, characterized by increased collagen content and a thicker fibrous cap, via the accumulation of SPCs (28). However, this study analyzed ligated carotid arteries, which display significant phenotypical differences in their lesion composition compared to ≪native≫ atherosclerotic lesions.Contrary, presence of CXCL12 does also induce recruitment of endothelial cell progenitors and fosters neovascularization in MI (72). While neovascularlarization in the context of MI seems beneficial another recent study has shown that active non-canonical NF-κB signaling in microvessels of carotid atherosclerotic lesions together with enhanced CXCL12 expresssion and neovascularization may induce plaque instability (73).

Overall, CXCL12 plays a critical role in the development of vascular remodeling as demonstrated by EC-derived CXCL12 mediating atheroprogression or the accumulation of VSMCs in the intima in response to increased CXCL12 titers (Figure 1) (26, 27, 69). Taken together, CXCL12 may serve as biomarker for CAD risk (70) and seems to drive macrophage foam cell formation and lesion growth. On the other hand, animal studies also suggest that it increases lesion stability by recruitment of SPCs and infiltration of medial VSMCs. Hence, therapeutical targeting of CXCL12 should only be cell specific and considered with great care.

### CXCL16

CXCL16 is an atypical chemokine containing a mucin-like stalk, transmembrane and cytoplasmic domains, which are not found in other CXC chemokines (74). CXCL16 has two distinct forms, the membrane-bound form which promotes the firm adhesion of cells expressing the receptor CXCR6 and the soluble form, generated by proteolytic cleavage of membrane-bound CXCL16, which acts as a chemoattractant for CXCR6^+^ cells (74). CXCL16 (Table 1) is expressed on stimulated ECs and VSMCs, macrophages, dendritic cells (DC) and platelets, whereas its receptor CXCR6 is expressed on memory and effector T cells, natural killer (NK) cells and NK T cells and is also found on plasma B cells (29, 75). CXCL16 is expressed in human atherosclerotic plaques and lesion severity is correlated with increased CXCL16 levels, as shown in human carotid endarterectomy specimens (76). It could be demonstrated that CXCL16 expression in the plaque enhanced platelet deposition on the endothelium (29). Furthermore, *in vitro* studies using human umbilical vein endothelial cells (HUVECs) established that the immobilization of CXCL16 promoted CXCR6-dependent platelet adhesion to the endothelium during physiologic flow and at low shear rates (77). This data implies that CXCL16 may play a role in vascular inflammation and thrombo-occlusive diseases. However, future investigations need to assess this relationship also on the more pathologically relevant arterial endothelium to understand platelet adhesion in an atherosclerotic model. Zhao et al. demonstrated that during ischemia reperfusion injury, cardiac EC-derived CXCL16 recruits CD11b^+^Ly6C^high^ inflammatory cells and facilitates the release of *tumor necrosis factor* α (TNFα) (interferon) IFN-γ and interleukin (IL)-17 in the heart. In line with this, silencing of CXCL16 by applying a specific shRNA reduced cardiac apoptosis, inflammation and dysfunction in ischemia reperfusion induced mice (78).

CXCL16 may also play a role in vascular remodeling through alteration of foam cell formation as its expression has been found to be upregulated in lipid-laden intimal macrophages and VSMCs (30). Moreover, CXCL16 may also directly contribute to foam cell formation as it is known to be a scavenger receptor for phosphatidylserine and oxLDL and its expression is upregulated in lipid-laden intimal macrophages and VSMCs via an autocrine mechanism (30). Growing evidence highlights the potential for VSMCs to develop into foam cells (71) and, like macrophages, VSMCs also express CXCL16 in response to IFN-γ stimulation, which also correlates with an increased uptake of oxLDL by VSMCs (31). Therefore, it can be surmised that CXCL16 facilitates the development of macrophage and VSMC foam cells (Figure 1). However, to our best knowledge so far no therapeutic treatment against CXCL16 has been investigated in the context of atherosclerosis and chronic vascular inflammation opening a potential interesting novel avenue of future research.

### CX3CL1

CX3CL1 also known as fractalkine is also a chemokine which can be present as a membrane-bound or soluble form (79). Both forms activate the chemokine receptor CX3CR1 (Table 1), where the transmembrane form induces integrin-independent leukocyte adhesion and the soluble form is a chemoattractant for leukocytes (80). During atherosclerosis, monocytes expressing CX3CR1 bind and adhere to the endothelium which express the membrane-bound form of CX3CL1 (81). CD16^+^CX3CR1^HIGH^ monocytes then activate endothelial *signal transducer and activator of transcription* 1 (STAT1), NFκB and p65 phosphorylation to upregulate proliferation and expression of CX3CL1, IL-1β, Intercellular adhesion molecule 1 (ICAM-1) and vascular cell adhesion molecule 1 (VCAM-1) by ECs (82, 83). Hence, the CX3CL1-CX3CR1 axis enhances a pro-atherosclerotic EC phenotype via the upregulation of adhesion molecules and inflammatory mediators. In this context, i*n vitro* studies have shown that CX3CL1 is upregulated on activated VSMCs and triggers monocyte adhesion to VSMCs (32). Further work has demonstrated that *Cx*3*cr*1^−/−^ animals—post femoral arterial injury induced *via* an angioplasty guide wire—were protected against intimal hyperplasia due to decreased monocyte trafficking to the lesion compared to the control group (34). In addition, CX3CR1 deficiency resulted in decreased VSMC proliferation and intimal accumulation, which is either directly or indirectly a result of defective monocyte infiltration (34). The relationship between CX3CL1-CX3CR1 and inhibited VSMC proliferation and intimal accumulation in vascular remodeling still needs to be elucidated in detail. One study has demonstrated that a mononuclear CX3CR1^+^ cell population residing in murine bone marrow provides a source of SPCs after wire-induced vascular injury, which differentiate into VSMCs within the neointimal plaque (35). Furthermore, BMT of CX3CR1 deficient bone marrow into C57BL6/J mice demonstrated that *Cx*_3_*cr*1 expression is essential for VSMC differentiation from SPCs in the vascular wall (35). Thus, CX3CL1 may play a prominent role in intimal hyperplasia promoting atherosclerosis through increased VSMC proliferation and monocyte trafficking. However, it would be worthwhile to perform similar studies using either an *Apoe*^−/−^or a *Ldlr*^−/−^ mouse fed a WD to provide results that truly reflect CVD triggered mechanisms.

Another recent study treated *Ldlr*^−/−^ mice with a CX_3_CL1-Fc fusion protein inhibiting the CX_3_CR1-CX3CL1 interaction. This fusion protein treatment significantly reduced atherosclerotic lesion size, independent of WD diet feeding and reduced M1-like macrophage and T cell accumulation in the aortic wall (33). Thus CX3CL1-Fc could be a potent therapeutic option interfering with vascular remodeling and atherosclerosis (Table 2). Along this line, another study implemented the use of a DNA vaccine, a vector that contains genes encoding a single-chain antibody specific for the mouse dendritic cell (DC) antigen DEC205 and the receptor CX3CR1 (DEC-CX3CR1) and a non-DC DNA vaccine (Con-CX3CR1). Both vectors were injected into *Apoe*^−/−^mice fed a normal chow diet (84). DEC-CX3CR1 vaccinated mice demonstrated a significantly reduced atherosclerotic plaque size compared to both Con-CX3CR1 vaccinated and unvaccinated mice (84). Furthermore, DEC-CX3CR1 mice showed reduced monocyte infiltration and lipid deposition in the lesions compared to unvaccinated mice, although the lesional macrophages still possessed an M1 phenotype. Not only does this further support the role of the CX3CL1-CX3CR1 axis in vascular remodeling (Figure 1), but it also emphasizes the need to conduct more studies on DNA vaccination (Table 2) as an effective therapeutic strategy against atherosclerosis and vascular remodeling. Overall, CX3CL1 plays an important role in M1 macrophage differentiation and the migration/proliferation of VSMC resulting in vascular changes (Figure 1).

## Cytokines

Cytokines are molecules that are secreted by immune cells and other specific cell types that modulate the inflammatory immune response and mediate cell-cell communication. Cytokines are subdivided into different classes: TNFs, IFNs, ILs, *transforming growth factors* (TGFs) including *growth/differentiation growth factors* (GDF), *colony-stimulating factors* (CSFs) and chemokines as detailed before. In atherosclerosis, pro-inflammatory cytokines play an important role in the initiation and progression of the disease and in the instigation of endothelial dysfunction, upregulation of adhesion molecules and promotion of immune cell migration as well as their infiltration into the lesion. All these different factors lead to arterial remodeling and subsequent changes in vascular function (85). The most important cytokines that are known to contribute to atherosclerosis lesion remodeling are IL-1, IL-6, IL-10, IL-22, IL-33, TNF-α and GDF-15 which will be discussed below in greater detail:

### Interleukin 1

IL-1 was one of the first cytokines to be discovered and it is divided into two related but functionally distinct isoforms: IL-1α and IL-1β. Here, the main focus will be on IL-1β as this is a potent driver of the inflammatory response in atherosclerosis and vascular inflammation. IL-1β is synthesized by many cells including neutrophils, NK cells, DCs, ECs macrophages, monocytes and SMCs (Figure 2). In atherosclerosis, its synthesis is triggered by the uptake of cholesterol crystals by macrophages activating the *NLR family pyrin domain containing* 3 (NLRP3) inflammasome or by the binding of IL-1 family members to their receptor IL-1R resulting in a positive autocrine inflammatory feedback loop (86). In addition to cholesterol, monocytic inflammasomes and the production of IL-1β can be activated by dying VSMC and in turn promotes VSMC proliferation as shown *in vitro* and *in vivo* in C57BL/6 mice of vein graft injury (87). IL-1β itself induces the upregulation of adhesion molecules such as ICAM1 and VCAM1 as well as the monocyte chemoattractant chemokine CCL2 (please see section CCL2) on ECs promoting leukocyte recruitment into the atherosclerotic plaque (Figure 1; Table 3) (88, 115, 116). Pro-inflammatory cytokine IL-1β has also an important role in cross-talk between ECs and the underlying VSMCs. Specific secretion of IL-1β by SMC induces E-selectin expression by ECs (117). In turn, IL-1β secretion by activated ECs promotes VSMC proliferative, synthetic and macrophage-like phenotypes (118). The barrier function of ECs is crucial to maintain vascular wall homeostasis. However, in atherosclerotic conditions, dyslipidemia and proinflammatory cytokines such as IL-1β promote excessive adhesion molecule expression by ECs leading to endothelial dysfunction and increased permeability of the EC barrier due to the disruption of intercellular junctions, resulting in increased leukocyte infiltration and vascular remodeling (Figures 1, 2; Table 3) (90). In addition, IL-1β promotes VSMC proliferation by stimulation of autocrine production of PDGF (Figure 1) and the production and release of the pro-inflammatory cytokine IL-6 (Table 3) (92, 93). IL-1β-induced EC dysfunction is also promoted by plasma *trimethylamine-N-oxide* (TMAO). TMAO is an oxidation product of the liver that is made from compounds synthetized by intestinal bacteria and an elevated concentration of TMAO increases monocyte mobilization and activation leading to low-grade inflammation (119). In line with this, high plasma levels of TMAO are associated with atherosclerosis and increased risk of CVD. In this context Boini et al. (119) showed that TMAO increases the assembly and activation of the NLRP3 inflammasome leading to an increased production of IL-1β in carotid arteries of WT mice with partially ligated carotid artery. In addition, *in vitro* experiments indicate that TMAO treatment induces NLRP3-dependent endothelial hyperpermeability by decreasing *zonula occludens-*1 (ZO-1), a tight junction protein responsible for junction integrity, expression in mouse carotid arterial endothelial cells (CAECs) (119). Therefore, targeting TMAO may help to reduce adverse remodeling in atherosclerosis by preventing EC leakage and infiltration of inflammatory cells as well as reducing IL-1β driven inflammation (119). IL-1β also increases the expression of *dipeptidyl peptidase* 4 (DPP4), which is a transmembrane protein expressed on ECs that is involved in glucose metabolism and cardiometabolic disease (120). Recent studies showed that DPP4 inhibition decreases atherosclerotic plaque burden. For example, Meng et al. investigated the atheroprotective role of the DPP4 inhibitor trelagliptin on HAECs exposed to IL-1β *in vitro*, showing that trelagliptin treatment had a strong inhibitory effect on the expression of adhesion molecules and pro-inflammatory chemokines and cytokines that orchestrate monocyte adhesion on the endothelium (89). Mechanistically, trelagliptin inhibits IL-1β induced NFκB transcription factor activation which subsequently prevents the transcription of the monocyte chemoattractant chemokines CCL2, CXCL1 as well as the pro-inflammatory cytokine IL-6 and the adhesion molecules ICAM1 and VCAM1 (mRNA and protein levels) (Table 3) (89).

**Table 3:** Overview of cytokines involved in atherosclerosis remodeling and their physiological effect.

**Cytokines**	**Receptors**	**Cells affected**	**Proposed effect in vascular remodeling**	**References**
IL-1β	IL-1R	ECs	EC dysfunction	(88)
			Expression of ICAM1, VCAM1, CCL2	(89)
			Leukocyte adhesion and infiltration into the intima	(90)
		Macrophages	Expression of IL-6, IL-8, TNFa, CCL2	(91)
		VSMCs	Proliferation	(92)
			IL-6 expression	(93)
			Collagen expression	
IL-6	Gp130	ECs	ICAM1, VCAM1 expression	(94)
				(95)
		Monocytes	Recruitment, infiltration	(96)
				(97)
		VSMCs	Recruitment and migration	(98)
				(95)
		Neutrophils	Recruitment	(96)
				(97)
		ECM	Collagen deposition	(99)
*IL-*10	*IL-*10*R*1	Macrophages	Prevent proinflammatory cytokines production	(100)
				(101)
				(102)
			Prevent foam cell formation	
	*IL-*10*R*2			
			*Promotes M*2 *macrophage polarization*	
IL-22	IL-1R1	ECs	ICAM1, VCAM1 expression	(103)
				(104)
				(105)
		Macrophage	Phenotypic change from anti-inflammatory into pro-inflammatory cell type, Reduced cholesterol efflux	(105)
		VSMCs	Migration and proliferation	(106)
			Phenotypic switch	(107)
			from contractile into synthetic repair cell	(105)
IL-33	ST2	ECs	VCAM1, ICAM1, E-selectin, CCL2 expression	(108)
		Macrophage	Inhibits foam cell formation	(109)
		T cells	Differentiation into Th2 cells	(109)
TNFa	TNF1 TNF2	ECs	ICAM1, VCAM1, CCL2 expression	(110)
		Monocytes	Recruitment, Differentiation into macrophages	(111)
				(112)
		Macrophage	Foam cell formation	(110)
		VSMCs	Proliferation	(113)
				(114)

Results from studies that focus on IL-β antibody blocking or knock out *in vivo* are less straightforward. Earlier work in *Apoe*^−/−^mice in which the anti-IL-1β antibody XMA052 MG1K was injected twice weekly during 16 weeks of WD showed a decrease of aortic lesion area of 37, 22 and 29% with 0.1, 1.0, 10 mg/kg XMA052 MG1K, respectively, compared to IgG injected control *Apoe*^−/−^ mice (91). *In vitro* experiments performed in the same study revealed reduced release of other pro-inflammatory cytokines such as IL-6, IL-8, TNF-α and CCL2 from cultured macrophages and reduced release of the proteolytic enzymes MMP-3 and MMP-9 from ECs and VSMCs, after XOMA 052, a human engineered IgG2 anti-IL-1β antibody, treatment (Tables 3, 4) (91). However, MMP-3 and MMP-9 expression were not affected *in vivo*, and no difference was observed in the plaque collagen content suggesting that plaque stability is not modified by this treatment (91). Yet, when anti-IL-1β treatment was tested to investigate its role in established atherosclerotic lesions, it led to adverse remodeling. Cell-specific effects of the IL-1β antibody (mouse monoclonal antibody, Novartis, 10 mg/kg) were tested by Gomez et al. on VSMC lineage tracing *Apoe*^−/−^ mice where the fate and migration of SMCs can be monitored during the development of atherosclerosis by using an inducible Cre-flox system to label MYH11^+^ SMC specific YFP expression (*Apoe*^−/−^*Myh*11-CreER^T2^R26R-YFP). The anti-IL-1β monoclonal antibody treatment did not lead to any differences in the aortic plaque size compared to IgG treated control animals after 18 weeks WD. In addition, anti-IL-1β treated mice showed thinner fibrous caps characterized by a 30% decrease of collagen, a 40% decrease of VSMC content, though a surprising 50% increase of lesional macrophages within the fibrous cap (Table 4) (121). These results indicate that anti-IL-1β treatment has a detrimental effect on fibrous cap remodeling promoting plaque instability.

**Table 4:** Targeting cytokines as therapeutic treatments in vascular remodeling and CVD.

**Cytokines**	**Therapeutic Treatment**	**Clinical trials**	**Animal experimentation**	**Outcomes on atherosclerosis**	**References**
IL-1 β	Canakinumab	✓	✓	Decreases aortic lesion area, Decreases IL-6, IL-8, TNFα, CCL2, Decreases collagen and VSMC content	(91)
					(121)
IL-6	Raloxifene	✓	✓	Decreases aortic lesion area, Decreases IL-6, Decreases ICAM1, VCAM1 expression, Decrease macrophage and VSMC content	(95)
	Ziltivekimab	✓	–	Decreases of serum CRP	(122)
	Tocilizumab	✓	–	Decreases CRP, Ameliorates FMD, Increase total cholesterol	(123)
IL-10	Nothing yet	–	–	–	–
IL-22	Fezakinumab	✓	–	Not published yet	(124)
IL-33	Nothing yet	–	–	–	–
TNFα	Adalimumab	✓	✓	Decreases VCAM1, E-selectin, CRP, and aortic stiffness; Increases oxLDL	(125)
					(126)
					(127)
	Etanercept	✓	–	Decreases aortic stiffness, Increases cholesterol and triglycerides	(128)

Nevertheless, the Canakinumab Anti-inflammatory Thrombosis Outcome Study (CANTOS) was the first big scale clinical trial confirming the potential of anti-inflammatory IL-1β therapy in CVD. In the CANTOS trial, anti-IL-1β (canakinumab) treatment resulted in a decrease of inflammatory markers in the plasma of patients such as high-sensitivity C-reactive protein (hsCRP) (26–41%) and IL-6 (6–25%) (129–131).

The receptor for IL-1β, *IL-*1β *receptor type* 1 (IL-1R1), was also described to reduce atherosclerotic plaque development (132, 133). However and in sharp contrast, lack of the *IL-*1β *receptor antagonist* (IL-1ra) promotes atherosclerotic plaque formation (134). More recent studies further support this controversial role of IL-1R1 and IL-1ra in atherosclerosis development and more precisely in atherosclerotic plaque remodeling.

IL-1R1 deletion has a dual role in plaque stability and outward vessel remodeling as shown in a female mouse model lacking IL-1R1 (*IL-*1*R*1^−/−^*Apoe*^−/−^) and fed a WD for 30 weeks. These mice showed reduced aortic root lesion size as well as a 50% reduction of the lumen area of the brachiocephalic artery when compared to control *IL-*1*R*1^+/+^*Apoe*^−/−^ mice. However, these reductions were accompanied by enhanced features of plaque instability, characterized by decreased VSMCs and collagen content. MMP-3 expression in the brachiocephalic artery was also significantly lower in *IL-*1*R*1^−/−^*Apoe*^−/−^ animals which may explain the reduced collagen content in the plaques (135). In line with this, IL-1Ra-deficient mice exhibited considerable inflammation in the aorta accompanied by an increased macrophage content in the adventitia and destruction of the elastic lamina, which is normally important to maintain arterial wall stability prevent intima lesion progression (136, 137). Another study also investigated the role of IL-1Ra in atherosclerosis lesion development by making use of *IL-*1*Ra*^+/+^ and *IL-*1*Ra*^+/−^ mice (*IL-*1*Ra*^−/−^ mice were excluded from this study due to reduced weight gain and differential cholesterol metabolism) on *Apoe*^−/−^ background. Heterozygous and control animals were fed normal chow diet for 16 weeks and *IL-*1*Ra*^+/−^*Apoe*^−/−^ mice showed a 30% increase of atherosclerotic lesion size compared to *IL-*1*Ra*^+/+^*Apoe*^−/−^ mice (134). Interestingly, after 32 weeks of chow diet feeding the lesion size was similar in both groups, but macrophage accumulation in the lesions was reduced in *IL-*1*Ra*^+/−^*Apoe*^−/−^ animals compared to control. VSMCs in the same lesions showed a 15% decrease compared to control *IL-*1*Ra*^+/+^*/Apoe*^−/−^animals (134). These findings suggest that IL1-Ra plays an important role in the development of atherosclerotic lesions and also suggests that it impacts plaque composition, modulation and stability (Figure 1).

Taken together, IL-1β is detrimental and promotes early-stage atherosclerosis development but on the other hand animal models suggest that it is beneficial in an advanced stage of the disease to maintain plaque stability and avoid major cardiovascular complications. Plaque stability also seems to be affected by interfering with IL1-R1 or IL-1RA. Nevertheless, it is important to keep in mind that research on mice and *in vitro* assays have certain limitations with regards to the translation to human pathologies. Therefore, efficacy and safety of the CANTOS trial and its promising beneficial results in patients with adverse cardiovascular events still hold true even if research on IL-1β on atherosclerosis in mice and *in vitro* findings are sometimes conflictual meaning that the results are not always consistent from one study to another. In addition, one should keep in mind that *Apoe*^−/−^ mice are severely hypercholesterolemic which does not fully translate with the CANTOS patients who were under statin treatment. Moreover, canakinumab in CANTOS was only administered quarterly and not weekly which may explain why no effects on lesion stability were reported yet. The latter also underlines the importance of careful timing of treatment regimens.

### Interleukin 6

IL-6 is secreted by various cells including epithelial cells, VSMCs, vascular ECs, monocytes, and T cells (Figure 2) (138) and promotes the synthesis of acute phase proteins such as CRP, amyloid A, β2 protein, hemopexin and haptoglobin. Both CRP and amyloid A are biomarkers of chronic inflammation and their levels predict cardiovascular risk (97, 139). In addition, IL-6 shows chemotactic activity for monocytes and neutrophils and promotes the expression of adhesion molecules (Table 3) (94) and chemokines leading to vascular remodeling by the induction of leukocyte adhesion and infiltration into the intima (Figures 1, 2; Table 3) (96, 97). More precisely, paracrine secretion of IL-6 by VSMC induces expression of the adhesion molecule E-selectin on ECs (117). In addition, in response to IL-6 signaling ECs and SMC also start to secrete IL-6 (140). TNFα activated ECs also induce SMC production of IL-6 (141).

Circulating IL-6 is a biomarker for CVD such as acute coronary syndrome with atherosclerosis (142). A study on carotid atherosclerosis showed that blood levels of IL-6 and TNFα were higher in patients with carotid atherosclerosis compared to control subjects and the cytokine levels increased with increasing amounts of carotid atherosclerosis stenosis (143). IL-6 was also shown to promote migration and viability of macrophages and VSMCs *in vitro* and regulate ECM deposition and reorganization (Table 3) (95, 98). One mechanism of IL-6 driven SMC migration is made via MyD88 and TRIF action and activation of p38 MAPK and ERK1/2 signaling) (144). Schieffer et al. investigated the role of IL-6 deficiency on atherosclerosis development. In this study, IL-6 deficient *Apoe*^−/−^ mice (*Apoe*^−/−^*IL-*6^−/−^) were fed with chow diet for 53 ± 4 weeks and showed a significant decrease in aortic transcript and protein levels of MMP-9, tissue inhibitor of metalloproteinase-1, collagen I and V and lysyl oxidase (145), an important protein for the formation and repair of the ECM, compared to *Apoe*^−/−^ control mice (146). Additionally, *Apoe*^−/−^*IL-*6^–/−^ mice exhibited a decrease in macrophage and leukocyte infiltration into the lesions, although atherosclerotic lesion formation was enhanced in *Apoe* ^−/−^*IL-*6^–/−^ compared to *Apoe*^−/−^ animals (28.1 vs. 14.9% respectively). The latter may be explained by the observed increase of total cholesterol, LDL, and very-low-density lipoprotein VLDL in IL-6 deficient animals and a decrease of plasma IL-10, compared to control mice (146).

Interestingly, a recent study showed an atheroprotective effect of raloxifene treatment in male *Apoe*^−/−^ mice fed a WD for 12 weeks. Raloxifene is a drug that is prescribed to post menopause women to prevent osteoporosis and is able to inhibit IL-6 binding to the IL-6 receptor subunit GP130, thereby prohibiting downstream signaling pathways such as STAT3. In this study, it was observed that daily administration of 5 mg/kg raloxifene reduced the area of atherosclerotic lesion size in the aorta and aortic root in *Apoe*^−/−^ mice compared with untreated control *Apoe*^−/−^ mice (95). In addition, raloxifene significantly decreased the expression of IL-6, ICAM1, and VCAM1 in the aortic vascular ECs and reduced the lesional macrophage and VSMC content in these aortic lesions. Mechanistically it could be demonstrated that raloxifene decreases atherosclerosis by preventing IL-6-induced phosphorylation of STAT3 and inhibiting the IL-6/GP130 interaction (Tables 3, 4) (95).

IL-6 secretion by aortic perivascular adipose tissue (PVAT) also promotes aortic stiffness and remodeling in *Ldlr*^−/−^ mice compared to WT C57BL/6 mice fed with chow diet (99). Aortic stiffness, mediated by changes in the ECM protein expression, is caused by an increase in cross-linking of collagen (147). Consistent with other studies, Du et al. (99) also observed an increase in IL-6-ediated collagen type I expression in the aorta of *Ldlr*^−/−^ mice (Table 3). These findings suggest that IL-6 may play an important role in inducing changes in the ECM by increasing collagen type I expression and thereby promoting subsequent arterial stiffness and thus vascular remodeling (Figure 1).

RESCUE, a trial to evaluate the reduction in inflammation in patients with advanced chronic renal disease utilizing antibody-mediated IL-6 inhibition investigated the effect of ziltivekimab in patients with high cardiovascular risk (122). Patients in this study had elevated serum CRP levels and chronic kidney disease and were randomly allocated into groups with differential monthly subcutaneous administrations of ziltivekimab (total of 7.5 mg, 15 mg or 30 mg) or placebo. After 12 weeks of treatment, CPR levels decreased by 77% in the 7.5 mg group and by 92% in the 30 mg group. Biomarkers of systemic inflammation and thrombosis, relevant to atherosclerosis, were also reduced in a dose-dependent manner, while no severe side effects were detected. Due to the COVID-19 pandemic, the study unfortunately had to be terminated before the planned secondary 24-week endpoint to avoid exogenous causes of increased CPR levels and thereby bias the interpretation of the results. However, it is planned to do a large-scale cardiovascular outcome trial on the same population to investigate the effect of ziltivekimab on the recurrence rate of vascular events (Table 4) (122). Another clinical study investigated the efficiency of an anti-IL-6 treatment, tocilizumab, on endothelial function in high-risk CVD patients with rheumatoid arthritis (RA). Tocilizumab is a humanized monoclonal antibody that targets the soluble and membrane-bound IL-6 receptor. The study consisted of three groups, whereby 18 patients received tocilizumab (8 mg/kg IV) every 4 weeks, 24 patients underwent anti-TNFα treatment (methotrexate 15–25 mg/week or leflunomide 20 mg/d) and 18 control patients who were treated with synthetic disease-modifying antirheumatic drugs (123). Endothelial function was evaluated by flow-mediated dilation (FMD) measurements before and after 16 weeks of starting the different treatments. As expected, patients from the tocilizumab treated group showed the most striking reduction in mean CRP levels compared to the other treatments. Furthermore, the mean FMD only significantly improved in the tocilizumab group, increasing from 3.43 to 4.96%. On the other hand, anti-IL-6 treatment greatly enhanced the atherogenic lipid profile and increased total cholesterol levels (Table 4) (123). Long-term treatment with tocilizumab was also addressed in a pilot study with 16 female RA patients receiving monthly injections with tocilizumab (8 mg/kg IV) and endothelial function was again measured by FMD. The endothelial function significantly improved after 6 months of treatment compared to 16 non-RA control subjects (148). As underlined by various clinical trials, tocilizumab worsens the lipid profile, total cholesterol burden, LDL, and triglyceride profile. However, it does also improve endothelial function and therefore reduces vascular remodeling. Therefore, to minimize the detrimental effects of elevated lipids, it would be ideal to for example combine tocilizumab with statin treatment.

### Interleukin 10

IL- 10 is an anti-inflammatory cytokine produced mainly by macrophages and T cells. IL-10 signaling is mediated through a two-receptor complex named IL-10 receptor 1 (IL-10R1) and IL-10R2. The receptor complex is constitutively expressed on numerous hematopoietic and non-hematopoietic cells such as epithelial cells and fibroblast and is upregulated upon activation (149). In atherosclerosis, IL-10 prevents remodeling by inhibiting macrophage activation and their proinflammatory cytokine production including TNFα, IL-1β, IL-6, IL-8, granulocyte stimulating factor (G-CSF) and granulocyte macrophage colony-stimulating factor (GM-CSF), foam cell formation, MMP expression, VSMC proliferation and therefore helps to reduce atherosclerotic plaque formation (Figure 1; Table 3) (101, 150).

In contrast, IL-10 deficiency in *Apoe*^−/−^ after 16 weeks on chow diet leads to greater atherosclerosis plaque formation, an increase in blood cholesterol (e.g., LDL), an increase in Th1 cell response in the lesion as well as greater tissue factor activities, systemic coagulation and vascular thrombosis compared to control (151). In APOE^*^3-Leiden mice under cholesterol-enriched high-fat diet with cuff-induced neointima formation in the femoral artery, IL-10 deficiency led to increased neointima formation. On the other hand, in the same mouse model, IL-10 overexpression by means of single intramuscular injection of IL-10 resulted in a 45% decrease of neointima surface (152). However, *Ldlr*^−/−^ mice transplanted with marrow cells from IL-10 transgenic male mice on a C57BL/6J background and fed chow diet for 4 weeks prevents the formation of advanced lesions, shifts Th1 cells toward Th2 phenotype and decreases IFN-γ levels in the lesion (153). Interestingly, IL-10 overexpression leads to increased modified LDL uptake by macrophages, although it also stimulates its efflux and thus prevents foam cell formation (Figure 1) (100). Overall, IL-10 diminishes lesion burden by decreasing proinflammatory cytokine levels and promoting an anti-inflammatory environment in the lesion with M2 polarized macrophages as well as Th2 cells and prevents foam cell formation (100, 154, 155). IL-10 also inhibits VSMCs activation *in vitro* and *in vivo*. IL-10 treatment of LPS-stimulated rat SMCs resulted in a decreased NF-κB activation, IL-6 secretion as well as reduced SMC migration and proliferation (Figure 1). In line with these observations, in a rat model of intimal hyperplasia, IL-10 treatment led to reduced SMC proliferation and intimal growth 14 days after balloon abrasion of the aorta compared with saline-injected control animals (150).

Furthermore, Jung et al. investigated more in detail the protective cellular and molecular mechanisms of IL-10 that prevent adverse MI LV remodeling. The authors show that IL-10 promotes M2 macrophage polarization *in vitro* and in turn the M2 secretome induces cardiac fibroblast activation, proliferation, migration and α-SMA expression. In addition, the same study showed that IL-10 treatment of fibroblasts reduces the ratio of collagen I to III secretion 7 days post-MI leading therefore to decrease fibrosis formation (Table 3) (102). These observations could mean that IL-10 levels may be implicated in plaque stability.

Systemic treatment with IL-10 is not ideal as it inhibits inflammation even when it is needed to fight pathogens. Indeed, long-term treatment of IL-10 increases the prevalence of intracellular infection such as Chlamydia and Listeria (156). In this regard, a study has tried cell-specific systems to avoid these off-target side effects. Exosomes loaded with IL-10 mRNA were engineered to target inflamed macrophages in the atherosclerotic lesion. These exosomes were used to treat *Apoe*^−/−^ mice under 8 weeks high fat diet and resulted in decreased atherosclerotic plaque formation compared to PBS receiving control or exosome treatment (157).

Treatments and clinical trials are currently undergoing to explore the anti-inflammatory properties of IL-10 in various chronic diseases including RA, multiple sclerosis, allergies and inflammatory intestinal disease among others. However, IL-10 therapy for atherosclerosis and its effect on vascular remodeling remains to be investigated.

### Interleukin 22

IL-22 is a member of the IL-10 cytokine family and is secreted by both innate and adaptive immune cells such as activated T cells especially T helper (Th) 22 cells and Th17 cells, NK cells, neutrophils, fibroblasts, and macrophages. IL-22 is involved in many cellular processes including lipid metabolism regulation, maintenance of bacterial homeostasis in the intestine and tissue regeneration (158).

The function of IL-22 in atherosclerosis is largely unknown, although evidence suggests that IL-22 is involved in vascular remodeling by promoting pro-inflammatory chemokines and antimicrobial peptide secretion as well as increasing VSMC migration and proliferation (Figure 1) (159, 160). In addition, IL-22 regulates adhesion molecule expression by ECs such as ICAM1 and VCAM1 as well as the production of chemokine ligands that have been implicated in adhesion, migration and recruitment of monocytes in atherogenesis (Figure 1) (103–105) (Table 3). The receptor of IL-22, being IL-22R1, is widely expressed on VSMCs, macrophages and ECs and mediates enhanced proliferation and migration through NFκB-, STAT3-, MAPK- and ERK1/2-dependent pathways in VSMCs (Figure 1). Furthermore, paracrine IL-22 promotes macrophage differentiation from anti-inflammatory into a pro-inflammatory phenotype and impairs the cholesterol efflux capacity of the cells, thereby promoting foam cell formation (Figure 1) (161, 162). Rattik et al. demonstrated that IL-22-deficient *Apoe*^−/−^ mice (*IL-*22^−/−^ *Apoe*^−/−^) fed a WD for 14 weeks had a significant reduction of atherosclerotic plaque size in both the aortic root and aorta, compared to control *Apoe*^−/−^ mice. In addition, *IL-*22^−/−^ *Apoe*^−/−^mice depicted reduced collagen content but increased expression of genes associated with VSMC contraction, namely α-actin, vinculin and caldesmon (Table 3) (106). The same study also explored the role of IL-22 in tissue repair mediated by arterial VSMCs in a carotid artery injury model in C57BL/6 mice. Here, an increased expression of IL-22 on VSMCs could be observed in the injured compared with non-injured arteries (106). These results suggest that IL-22 plays a key role in atherosclerotic plaque formation via the stimulation of dedifferentiation of contractile VSMCs toward synthetic repair cells resulting in plaque growth and that IL-22 is involved in plaque stability by thickening the fibrous cap, rendering it more stable by promoting lesional collagen content (Figure 1). IL-22 is therefore a double-edged sword. On the one hand, IL-22 worsens atherosclerosis by promoting inflammation, dysregulating macrophage cholesterol metabolism leading to more foam cell formation and promoting VSMCs proliferation and fibrous cap thickening (Table 3) (105). While on the other hand, IL-22 also leads to more stable plaques by increasing VSMC proliferation and migration into the intima forming a thick fibrous cap which decreases the risk of rupture and acute cardiovascular events.

Besides the above described protective effect of IL-22 it was also demonstrated that this cytokine can prevent atherosclerosis by promoting the expression of antimicrobial peptides which limit the spread of proatherogenic bacteria such as *Enterobacteriaceae* (*Klebsiella* sp)*, Prevotellaceae* (*Prevotella copri*), *Lachnospiraceae, Clostridiaceae* and *Ruminococcaceae*. Additionally, a BMT of *IL-*22^−/−^ BM into *Ldlr*^−/−^ mice fed with WD for 16-weeks revealed larger atherosclerotic lesion sizes in hematopoietic IL-22-deficient *Ldlr*^−/−^ mice compared to the control group (158). In these mice the aortic plaque was characterized by an increase of T cells and myeloid cell content as well as enhanced expression of aortic and intestinal proinflammatory cytokines like IL-1β, TNFα, CCL2 and CCL5. Intestinal gene expression of anti-microbial peptides *C-type regenerating islet derived-*3 (Reg3)-b and Reg3-g were also significantly reduced in hematopoietic IL-22 deficient *Ldlr*^−/−^ mice compared to controls and whole metagenome shotgun sequencing analysis of cecal luminal microbiota revealed an increase of the above mentioned pro-inflammatory and proatherogenic bacterial species in absence of IL-22 (158).

However, in sharp contrast, more recently Shi et al. (107) underlined the pro-atherogenic effects of IL-22 produced by Th22 cells. After 12 weeks of WD feeding while *Apoe*^−/−^ mice were treated with intraperitoneal injections of 2 μg recombinant mouse IL-22 (rIL-22) three times a week, it could be shown that IL-22 treatment resulted in larger atherosclerotic plaque sizes in the aortic root and the aorta as well as an increase in lesional macrophages. Th22, Th17 cells as well as DCs, collagen and serum IL-6 levels were also enhanced compared to PBS-treated *Apoe*^−/−^ control mice. In addition, SMC α-actin was reduced in mice undergoing rIL-22 treatment. These observations were abolished when mice were treated three times a week with 20 μg anti-IL-22 monoclonal antibody (IL-22 mAb), proving the causal role of IL-22 in the observed effects. The same study also showed that BM-derived DCs from *Apoe*^−/−^ mice treated with 100 ng/mL rIL-22 followed by stimulation with oxLDL displayed enhanced maturation properties and were able to induce differentiation and proliferation of naïve CD4^+^T cells into Th17 cells (107). The authors concluded that IL-22 secretion by Th22 aggravates atherosclerosis by promoting T cells (Th17), DCs and macrophage infiltration in the plaque and by inducing the dedifferentiation of contractile SMCs into synthetic SMCs (Table 3).

Human studies have also observed a correlation between circulating IL-22 levels and atherosclerosis. 45 patients with carotid artery disease were selected and were classified as symptomatic or asymptomatic based on the presence or absence of cerebrovascular symptoms. Immunostaining of plaques revealed a 7.15-fold higher IL-22 occurrence in symptomatic patients compared to the asymptomatic ones (104). Moreover, significant higher plasma levels of IL-22 were measured in patients with acute MI compared to healthy controls (163). Pre-clinical and clinical studies testing fezakinumab, a human anti-IL-22 mAb, and mAbs targeting the IL-22 receptor are still ongoing in patients with severe inflammatory diseases such as psoriasis, atopic dermatitis and rheumatoid arthritis. The limited results that are already published suggest no adverse safety concerns and <50% skin improvement, based on Percentage Change in the Scoring of Atopic Dermatitis (SCORAD) scores in atopic dermatitis patients (Table 4) (124, 164). Since atherosclerosis is also an inflammatory disease, interfering with IL-22 or IL-22 receptor may also represent a promising therapeutic target for CVD, although more elaborate research is needed to pinpoint the exact mechanisms of action.

### Interleukin 33

IL-33, the most recently discovered member of the IL-1 cytokine family may also play a crucial role in vascular remodeling. This cytokine is expressed by ECs, VSMCs, epithelial cells and immune cells such as macrophages and T cells. For example, IL-33 is released from injured or necrotic ECs and acts as an alarmin leading to pro-inflammatory responses, both from innate and adaptive immune cells (165, 166). IL-33 binds to *IL-*1 *receptor like protein* (IL-1RL1 or also known as ST2). ST2 has two forms, a transmembrane (ST2L) and a soluble (sST2) one, which compete with each other for IL-33 binding. ST2L is expressed by various immune cells such as macrophages, T cells (predominantly Th2), mast cells, and innate lymphoid cells (167). Elevated levels of IL-33 and its soluble receptor sST2 were observed in patients suffering from pathologies such as diabetes, obesity, CAD, stroke and atherosclerosis (168).

IL-33 promotes the expression and secretion of various pro-inflammatory cytokines, adhesion molecules, proteolytic and coagulation factors. Investigations of IL-33 in atherosclerosis revealed that this cytokine stimulates the expression of the endothelial adhesion molecules VCAM1, ICAM1 and E-selectin as well as the expression of CCL2 in HUVECs in a concentration-dependent manner resulting in increased leukocyte adhesion (Figure 1; Table 3) (108). On the other hand, by binding to the ST2L, IL-33 can also influence the phenotype and function of macrophages and T cells (167). Furthermore, Miller et al. (109) found that IL-33 reduces atherosclerotic lesion size in the aortic sinus of 6-week-old male *Apoe*^−/−^ mice fed with WD for 12 weeks while treated twice weekly with IL-33 injections (1 μg/injection) compared to the PBS-injected control group. In the same study, it could be demonstrated using *in vitro* serum assays that the atheroprotective effect of IL-33 was due to IL-33-mediated polarization of T cells into Th2 cells, inhibition of foam cell formation and an increase of antibody production targeting oxidized LDL. Nevertheless, plaque stability from the treated and untreated group remained unchanged, characterized by similar VSMC and collagen content, suggesting that IL-33 treatment decreases atherosclerosis plaque size without affecting plaque stability (Table 3) (109).

However, also some controversial results regarding the effects of IL-33 were found in human and rat studies. In humans, circulating IL-33 levels were found to correlate with vulnerable and high-risk plaques in 191 patients with carotid artery atherosclerosis. Analysis of carotid endarterectomies (CEA) from these patients also revealed an increased plaque expression of IL-33 and its receptor ST2 as well as enhanced IL-33 serum levels in CEA patients with vulnerable plaques compared with CEA patient with stable plaques (166). In contrast to what was observed in humans, obese rats showed increased sST2 levels in the aorta which correlated with an increase in the production of collagen, fibronectin and profibrotic molecules enhancing ECM formation and vascular fibrosis (Figure 1) (169). IL-33 seems to have differential effects on the stability of the atherosclerotic plaque depending on the species, in humans it was associated with vulnerable plaques however in rats, IL-33 seems to increase plaque stability.

In addition, it was found that certain genetic polymorphisms in the IL-33 locus resulted in deferential atherosclerosis development in patients with RA. From 576 RA patients carrying the mutant TT genotype of IL-33 rs3939286 polymorphism had a significantly lower carotid intima-media thickness (cIMT) evaluated by carotid ultrasound, compared to the wild-type CC genotype. The heterozygous CT genotype had an intermediate cIMT value. Combined, these results suggest a potential protective effect of the IL-33 rs3939286 T allele in atherosclerosis development by decreasing IL-33 expression (170).

However, there are also studies showing that atherosclerosis severity was not affected by IL-33/ST2 signaling. For example, there were no significant differences in atherosclerotic lesion area between *IL*33^−/−^*Apoe*^−/−^, *ST*2^−/−^*Apoe*^−/−^ or *Apoe*^−/−^ mice fed with 10-weeks-high cholesterol diet (171).

In conclusion, research on IL-33 and its multiple controversial effects on atherosclerosis are not yet fully elucidated and need to be better understood. A potential explanation for the controversial results are differences in tissue specific expression patterns, the applied model (e.g., deficiency vs. receptor blocking) or the prescribed medication, which should be investigated and compared more closely in future studies.

### Tumor Necrosis Factor α

TNFα is a pro-inflammatory cytokine that is expressed mainly by macrophages, DCs and T cells and activates various pathways including cell survival, apoptosis, necrosis, migration, proliferation, barrier disruption, cell adhesion and actin cytoskeleton modification (172). It has two receptors TNFR1 and TNFR2, although several studies showed that the majority of TNFα signaling is mediated through TNFR1 (173–175). TNFα modulates vascular remodeling by increasing EC permeability, up-regulation of endothelial adhesion molecules leading to monocyte adhesion to the endothelium, matrix degradation and VSMCs proliferation in the intima (Figure 1; Table 3) (111, 113, 176, 177). TNFα can act both in a paracrine or autocrine manner. For example, TNFα-activated ECs stimulate VSMC expression of the pro-inflammatory cytokine IL-6 but also vascular endothelial growth factor (VEGF) and further TNFα expression (141). Furthermore, TNFα secreted by ECs leads to VSMC proliferation as well as promoting synthetic and macrophage-like phenotype differentiation (118).

Therefore, TNFα is a key atherosclerotic cytokine in atherosclerosis (178) and its genetic deletion in atherogenic *Apoe*^−/−^ mice fed 10 weeks of WD was found to decrease atherosclerotic lesion size by 50% compared to control *Apoe*^−/−^ animals (112). In the same study a BMT of 10-week-old *Apoe*^−/−^ mice with age-matched *Apoe*^−/−^*TNF*α^−/−^ BM resulted in a 83% reduction of lesion size after 25 weeks of WD in mice with hematopoietic TNFα-deficiency (112). Unexpectedly, no differences in lipid burden, VCAM1 expression, macrophage, B cell and T cell numbers in the circulation could be observed comparing TNFα-deficient mice with control animals. These results suggest that hematopoietic TNFα may not be involved in monocyte mobilization, since adhesion molecules as well as macrophage content in the plaques were similar in both groups (Table 3) (112). Another study using *Apoe*^−/−^*TNF*α^−/−^ mice found a decreased expression of the adhesion molecules VCAM1 and ICAM1 and the cytokine CCL2 in TNFα-deficient mice on chow diet compared to *Apoe*^−/−^ control mice (Table 3) (110). The same study also described an reduced capacity of macrophages to phagocytose LDL particles, thereby promoting foam cell formation as well as an decreased expression level of the scavenger receptor class A in TNFα-deficient *Apoe*^−/−^ mice compared to *Apoe*^−/−^ controls (110) (Figure 1; Table 3). Taken together, it seems that somatic TNFα deficiency has a greater impact on atherosclerosis development as it decreases endothelial adhesion molecule expression as well as foam cell formation compared to hematopoietic TNF-α deficiency.

Anti-TNFα therapies in RA patients are also known to decrease the level of serum chemerin, an adipokine that has an important role in CVD, although the relationship of chemerin and TNFα in remodeling remains unknown (179). Nevertheless, vascular remodeling seems to be affected by chemerin levels as shown *in vitro*, where chemerin deficiency decreases VSMCs proliferation and *in vivo* where it decreases neointima hyperplasia after angioplasty (180). Lack of chemerin also leads to the reduction of pro-inflammatory cytokines, like TNFα, in the serum suggesting a feedback loop between the two (179). Another component that modulates TNFα-induced remodeling is the *erythropoietin-producing human hepatocellular receptor* (EphA), which is a receptor tyrosine kinase that mediates cell-adhesion and leukocyte homing in atherosclerosis by promoting ICAM1 and VCAM1 expression on ECs (181). EphA2-deficient *Apoe*^−/−^ mice which were fed a WD for 12 weeks developed smaller innominate artery and carotid sinus plaque size compared to *Apoe*^−/−^control animals (182). Furthermore, *in vitro* knockout of EphA2 in HAECs revealed that TNFα treatment is unable to induce monocyte adhesion to ECs lacking EphA2, suggesting that the TNFα-induced expression of adhesion molecules is inhibited upon EphA2 deficiency (182).

The effect of TNFα on endothelial function may also be modulated by food consumption. For example, aged garlic extract (AGE) and its sulfur-containing constituents improve the endothelial barrier function elicited by TNFα through stimulation of anti-inflammatory, anti-oxidative and anti-hypersensitive pathways in humans thereby also preventing CVD development including atherosclerosis. Active substances in AGE consisting of *S-*1*-prpenylcysteine* (S1PC) particularly interfere with TNFα-induced hyperpermeability of the endothelium (183). Therefore, adverse remodeling in atherosclerosis may also be reduced by food supplements.

Furthermore, tongxinluo (TXL), a traditional Chinese medicine product has anti-inflammatory as well as vasoprotective properties. In C57BL/6 mice subjected to carotid artery ligation, TXL treatment significantly reduced neointima hyperplasia in a dose-dependent manner by inhibiting macrophage infiltration as well as VSMCs proliferation in the intima of the artery, compared to untreated control mice (114). Further analysis revealed that the protective effects of TXL in hyperplasia are due to the inhibition of TNFα-induced miRNA-155 expression, a generally pro-inflammatory acting miRNA (114).

Nevertheless, until recently the therapeutic potential of TNFα blockage in atherosclerosis by pharmacological inhibitors such as monoclonal antibodies remained unknown. Oberoi et al. tested weekly injections of mouse-specific anti-TNFα monoclonal antibody CNTO5048 (12 mg/kg) in 10-week-old *Ldlr*^−/−^ mice fed with high fat, high cholesterol diet for either 6 or 12 weeks. Plasma inflammatory markers such as IL-6, CCL2 and TNFα were significantly decreased in mice receiving CNTO5048 after 12 weeks of treatment compared to control animals injected with IgG antibody (184). However, no differences were observed in the 6 weeks group, although mRNA expression levels of IL-6, CXCL1 and ICAM1 in vascular tissue of the aortic arch were increased after both 6- and 12-weeks treatment. On the other hand, plasma lipid profiles revealed a significant increase in VLDL cholesterol and triglycerides due to CNTO5048 treatment. Moreover, atherosclerotic plaque burden was also augmented in CNTO5048 mice. Detailed examination of the plaque composition revealed a reduction of intimal VSMC infiltration and lower collagen type I deposition within the atherosclerotic plaque in the CNTO5048-treated group which is associated with plaque instability. These counter-intuitive results revealing reduced systemic inflammation though enlarged lesion growth are most likely due to the enhanced cholesterol levels observed in the treated group. Yet, analysis of genes regulating lipid and cholesterol metabolism, such as *Apob, Mttp* or *Apoa*5 in the liver, did not reveal any differences between the two groups (184). In contrast, adalimumab, a human-specific anti-TNFα monoclonal antibody binding and blocking both soluble and membrane bound TNFα, administered to *Ldlr*^−/−^ mice fed 10-weeks of WD, revealed reduced atherosclerotic lesion size by 52% in the anti-TNFα treated mice (2.2 mg/kg twice weekly *via* intraperitoneal injection) compared to IgG controls, while the cholesterol and triglyceride levels did not change between the groups (185). These differential results may be due to the different antibodies that were applied, since the mouse model and length of WD feeding were comparable between the two studies (184, 185).

Various clinical trials have already been conducted to evaluate the effect of anti-TNFα treatment in patients with CVD. For example, recent findings reveal a decrease in cardiovascular biomarkers, such as soluble VCAM1, in patients after treatment with adalimumab. Psoriasis is well known to increase CVD risk as well as sharing different biomarkers and pathophysiological mechanisms such as systemic inflammation and endothelial dysfunction with CVD, suggesting that this pathology might benefit from similar therapeutic approaches (186, 187). Therefore, Zdanowska et al. conducted a study using 34 patients with psoriasis and 8 healthy volunteers between 30 and 73 years old, which were treated with an initial dose of 80 mg and then 40 mg of adalimumab every 2 weeks. After 12 weeks of treatment, soluble VCAM1 serum levels were significantly decreased comparing psoriasis patients with controls, while E-selectin was not affected (Table 4) (125). On the other hand, E-selectin levels were decreased in a bi-yearly study examining the effect of adalimumab in 17 patients with psoriasis compared with 24 healthy age-, gender- and BMI-matched volunteers with the same treatment regimen. Serum levels of E-selectin as well as plasma levels of IL-22 were significantly decreased compared to baseline, both after 12 and 24 weeks of treatment (126). Plasma CPR levels also decreased but only reached a significant difference compared to baseline after 24 weeks of treatment. Notably and in sharp contrast to the previously described beneficial outcomes, circulating oxLDL levels increased during the 2 years follow-up (Table 4) (126). The atheroprotective effects of adalimumab may therefore be a cumulative effect from a repression of inflammation and reduction of cholesterol uptake by macrophages leading to reduced foam cell formation. However, since cholesterol uptake by macrophage is decreased, it causes an augmentation of circulating cholesterol, which is a side-effect that could be solved with a combinational treatment with statins.

In addition, adalinumab decreases aortic stiffness as tested in 18 RA patients receiving either a monotherapy of subcutaneous adalinumab (40 mg/ 2 weeks) or a combination with disease modifying antirheumatic drugs in comparison to control patients using methotrexate (MTX). After 3 months of therapy, adalinumab treatment significantly decreased the aortic stiffness indices as measured by carotid-femoral pulse wave velocity (cfPWV) from 8.18 m/s to 7.01 m/s while no difference was observed in patients using MTX. On the other hand, there was no significant difference in the disease augmentation index (AIx), a systemic arterial stiffness parameter (188), or other cardiovascular risk factors after the adalinumab treatment (Table 4) (127). Besides adalinumab, also another TNFα inhibitor called etanercept is used as therapy for patients with various chronic inflammatory diseases such as RA. Based on a recent meta-analysis, both treatments significantly decreased vascular remodeling by limiting aortic stiffness and wave reflection in RA patients, independently of the treatment duration (duration varied from 6 to 56 weeks) (Table 4) (128). However, in all the studies mentioned above, TNFα treatment led to enhanced cholesterol and triglyceride titers (Table 4).

In conclusion, TNFα therapy holds great promise in reducing CVD and benefitting cardiovascular patients by decreasing atherosclerotic plaque size as well as decreasing several pro-inflammatory markers and ameliorating aortic stiffness. However, the long-term effects of anti-TFNα treatment and augmentation of cholesterol levels need to be critically considered when judging on the superiority of the treatment for CVD patients in light of the unfavorable lipid profile (128). However, based on clinical studies, the increase in lipid profiles in patients undergoing TNF-α treatment could be reduced by combining it with statin treatment (189).

## Other Mediators

In addition to chemokines and cytokines also other factors influence vascular function and composition. Among them are for example growth factors, prostaglandins and leukotrienes which will be summarized with respect to their role in vascular remodeling in the following paragraphs.

### Growth Differentiation Factor-15

*Growth differentiation factor-*15 (GDF-15), also known as macrophage inhibitory cytokine-1, is a member of the TGF-β family which is induced under stress conditions such as oxidative stress, inflammation, ischemia, or mechanical stretch (190). GDF-15 is known to be expressed in cardiomyocytes, especially in response to MI, but also by adipocytes, macrophages, ECs, and VSMCs from both healthy and injured tissues (191). A receptor for GDF-15 has been identified called *glial cell-derived neurotrophic factor* (GDNF) *family receptor* α*-like* (GFRAL). However, this receptor is mainly expressed on human brain stem cells and based on the various physiological effects of GDF-15 throughout the body it is hypothesized that other receptors should exist which are still to be discovered (192). Inflammatory proteins such as IL-1β, TNFα, IL-2 and *macrophage stimulating factor* (MCSF)-1, all of which are upregulated in atherosclerosis, induce the expression of GDF-15 (192). In line with this, an increase in circulating GDF-15 protein was recently associated with an elevated risk of adverse events in patients suffering from acute coronary syndrome, chronic kidney disease or heart failure (193–197). For example, the study by Gohar et al. (194), demonstrated that high plasma levels of GDF-15 in women with carotid atherosclerotic disease are predictive for secondary cardiovascular events. However, the effect of GDF-15 on cardiac remodeling is still poorly understood. One study from Xu et al. (198) showed that GDF-15 blocks norepinephrine-induced myocardial hypertrophy through a novel pathway involving the inhibition of epidermal growth factor receptor (EGFR) transactivation. In line with this observation, a recent overview from Wesseling et al. (199) emphasizes the role of GDF-15 in endothelial dysfunction, hypertrophy, and fibrosis (Table 3). GDF-15 was also associated with an elevated risk of adverse events in patients suffering from coronary syndrome, chronic kidney disease or heart failure (199). Heart failure involves cardiac remodeling following tissue injury which is caused by inflammation, volume, or pressure overload (199).

Growing evidence indicates that GDF-15 has detrimental effects on endothelial function by causing an increase in adhesion molecule expression and influencing the balance between vasoconstriction and vasodilatation (Figure 1). Indeed, GDF-15 leads to endothelial dysfunction by reducing vascular contraction and relaxation, which ultimately leads to an impaired cardiac function (Table 3) (190, 200). In addition, GDF-15 is implicated in cardiac hypertrophy which is described as an increased heart size and insufficient cardiac output (199). The exact role of GDF-15 on cardiomyocytes is still not fully understood but it seems that GDF-15 has both pro-hypertrophic and anti-hypertrophic effects depending on environmental cues. For example, GDF-15 promotes cardiac hypertrophy by protecting cardiomyocytes from apoptotic stimuli (201), but in constrast GDF-15 also seems to reduce myocardial hypertrophy by inhibiting the transactivation of EGFR (Table 3) (198). Further research also suggests that the anti-hypertrophic effects of GDF-15 on cardiomyocytes may be due to GDF-15-induced activation of *small mother against decapentaplegic* (SMAD) 2/3 proteins (202). GDF-15 also promotes cardiac fibrosis in heart failure and increases collagen turnover in MI as well as collagen deposition in atherosclerotic plaques (Figure 1; Table 3) (203–205).

In addition, elevated circulating GDF-15 levels are suggested as a potential biomarker for cardiovascular risk and outcome as it is directly linked to atherosclerosis progression. GDF-15 promotes plaque vulnerability through pro-inflammatory and angiogenic effects, especially in the early stages of the disease (205, 206). Furthermore, GDF-15 has been linked to cardiovascular event prediction in general and identification of high-risk patients (205, 206). Therefore, monitoring and targeting GDF-15 in CVD may improve early diagnosis and treatment strategies (206).

### Prostaglandins

Prostaglandins are a group of lipids synthesized at sites of tissue damage or infection and play an important role in resolving injury and illness by regulating inflammation, blood flow and the formation of blood clots (207). The production of prostaglandins is initiated by *cyclooxygenase* (COX) which induces the production of *thromboxane A*2 (TXA2) and prostaglandins, such as *prostaglandin* (*PG*)*-D*2, PG-I2, PG-E2 and PG-F2α (208). TNFα induces the expression of the prostaglandin endoperoxide synthase COX2 in a variety of cell types including ECs and VSMCs (186, 209, 210). PGs have also been found to induce diverse effects on VSMCs. For example, PG-D2 dictates the balance of VSMC proliferation and apoptosis and PG-E2 causes VSMCs to contract by inhibiting the potassium current (211, 212). PG-E2 also regulates VSMC tone through the *prostaglandin E* 1 (EP-1) and EP-3 receptors. Furthermore, EP-1 and EP-3 activation mediates intracellular Ca^2+^ pathway activation and the reduction of cAMP induced vasoconstriction (213). In sharp contrast, PG-E2 stimulation of EP-2 and EP-4 increases cAMP promoting vasodilation (214). Moreover, stimulating contractile VSMCs with PG-D2 activates the ERK pathway resulting in PG-D2 dependent VSMC phenotypic switching (215, 216). In CVD animal models, e.g. in *microsomal prostaglandin E*2 *synthase-*1^−/−^ (*mPGES*1^−/−^) *Ldlr*^−/−^mice on a 3- and 6-month WD, PGES1-deficiency, resulting in reduced circulating PG-E2 levels and resulted inreduced plaque burden and foam cell accumulation without affecting the blood pressure (217). PG-D2 is also induced by TNFα and, in turn, propagates the dedifferentiation of contractile VSMCs into a synthetic phenotype via the upregulation of *proliferator-activated receptor* (PPAR) (218). Furthermore, the loss of TNFα or the inhibition of COX2 repressed the induction of intimal thickening in mice (218). The same study also found that TNFα stimulates the activity of COX2 and thereby enhances COX2 expression in contractile VSMCs, while inhibition of COX2 suppressed TNFα-induced contractile VSMC phenotypic switching and neointima formation (218). Taken together, PGs have a diverse role in vascular remodeling (Figure 1), from regulating vasoconstriction and vasodilation to VSMC proliferation and apoptosis. Yet, specifically in atherosclerosis PGs like PG-E2 can dictate lesion size (217) and PG-D2 plays an important role in the phenotypic switching of VSMCs to their pro-atherosclerotic synthetic phenotype (218). However, it is difficult to suggest PGs as a potential therapeutic target as COX2 selective inhibitors are associated with increased atherothrombotic risk (219). Therefore, further research is needed to better understand the role of PGs in vascular remodeling before developing specific treatment approaches.

### Leukotrienes

Leukotrienes (LTs) are biologically active molecules produced by leukocytes, mastocytoma cells and macrophages in response to immunological and nonimmunological stimuli. LTs are well known as allergic, acute and chronic inflammatory mediators and are involved in several inflammatory conditions such as human arthritis, asthma, allograft rejection and atherosclerosis (220, 221). These inflammatory LTs originate from the 5-lipoxygenase (5-LO) pathway of arachidonic acid metabolism and, together with the 5*-LO-activating protein* (FLAP), catalyze the arachidonic acid metabolism from membrane phospholipids resulting in the formation of LT-A4, which is an unstable precursor leukotriene. 5-LO can further be metabolized into LT-B4 or form cysteinyl-leukotrienes like LT-C4, -D4 and -E4 after conjugation with glutathione (222, 223). Macrophages are the main producers of 5-LO and a correlation has been found between macrophage content of 5-LO and 5-LO localized in DCs, foams cells, mast cells and neutrophils and atherosclerotic plaque size in humans (224). In addition, clinical, population genetic, cell biological and mouse studies have all linked the 5-LO pathway to atherogenesis and arterial wall remodeling (223). In atherosclerosis, LTs promote the migration and accumulation of inflammatory cells into the intima of the vascular wall resulting in the initiation and progression of the disease (Figure 1). In addition, the inflammatory response is enhanced by the activation of leukotriene B_4_ receptors 1 (BLT_1_) and 2 (BLT_2_) as well as the *cysteinyl-leukotrienes receptors* (CysL)-T_1_ and CysLT_2_.These receptors are expressed on immune cells and vascular cells which are associated with atherogenesis such as ECs, VSMCs and monocytes/macrophages and their activation through leukotriene binding lead to structural alterations of the vascular wall and thus vascular remodeling (222, 223).

Direct effects of LTs on blood vessels include left ventricular contractility modifications, blood pressure regulation, coronary artery contraction and leukocyte recruitment into the perivascular space (225–228). *In vitro* studies revealed that LT treatment of ECs enhances the surface expression of P-selectin, secretion of von Willebrand factor and stimulates the synthesis of platelet activation factors and promotes VSMCs proliferation (229–231). LT-B4 is expressed on diverse cell types and for example neutrophils, eosinophils, VSMCs and monocytes all respond to LT-B4-dependent cell migration and recruitment to sites of inflammation (Figure 1) (220, 228, 232). In addition, the LT-B4-BLT1 pathway mediates VSMC recruitment and proliferation in the atherosclerotic plaque leading to intima hyperplasia as shown in rats treated with the BLT receptor antagonist BIIL 284 (10 mg/kg, once daily for 14 days) after balloon-induced injury of the carotid artery (233). Overall, it is clear that LT-B4 plays an important role in leukocyte attraction and adhesion to the vascular endothelium and the proliferation and migration of VSMCs (Figure 1) (231, 234).

As 5-LO is the regulator of the production of LT-B_4_ and cysteinyl leukotrienes, it is an interesting therapeutic target. 5-LO inhibition with intake of 0,1% BHB-TZD (5-(3,5-di-tert-butyl-4-hydroxybenzylidene)thiazolidin-2,4-dione) mixed in food was described to prevent plaque progression in atherogenic *Ldlr*^−/−^ mice fed a WD for 8 weeks (235). Moreover, 5-LO inhibition with licofelone in rabbits significantly decreased the femoral artery intima/media ratio as well as macrophage infiltration in the neointima, CCL2 expression and the activation of NFκB in the vascular lesion (236). Therefore, licofelone seems to diminish neointima formation following arterial wall injury and reduces inflammatory cell recruitment and adhesion into the arterial wall, thereby reducing atherosclerosis vascular remodeling.

Targeting LT receptors may represent an additional putative therapeutic target for the treatment of atherosclerosis and for preventing intimal hyperplasia after angioplasty. The *Carotid Atherosclerosis Progression Study* (CAPS) investigated 8 genetic polymorphisms associated with the leukotriene pathway and early atherosclerosis and remodeling in 969 patients. However, no significant effect of these polymorphisms was observed on atherosclerosis and remodeling risk based on carotid intima-media thickness (237). This result may be explained by the fact that most patients only showed signs of early atherosclerosis, while there was insufficient plaque advancement and stenosis to demonstrate associations with advanced atherosclerosis. Another randomly sampled cohort of 470 healthy, middle-aged women and men from the *Los Angeles Atherosclerosis Study* (LAAS) investigated the association between 5-LO gene promoter polymorphism, dietary arachidonic acid intake and the effect on atherosclerosis. An increase in intima-media thickness and atherosclerotic plaques could be observed in patients carrying two variant alleles of the 5-lipoxygenase compared with patients with the common allele. In addition, among the persons with the two variant alleles, the ones ingesting more arachidonic acid had significantly elevated intima-media thickness compared to patients with a marine n-3 fatty acid rich diet. These findings suggest a diet-gene interaction effect which impacts the development of atherosclerosis (238).

An ongoing clinical study (started in May 2020, estimated end in December 2023) is examining the role of a cyteinyl leukotriene antagonist in atherosclerosis (NCT04277702). They aim to study its effect on lower limb artery re-occlusion rate in 200 patients with peripheral artery disease after endovascular treatment. Results will further demonstrate whether LTs are a promising therapeutic option.

## Conclusion

Taken together many mediators exert divers and, in some cases, even opposing functions in atherosclerotic vascular remodeling. Future studies are needed to demonstrate whether the effects of CCL2, CCL5, CCL19, CXCL12, CXCL16 and CX3CL1 on VSMCs like increased proliferation and subsequent increase of plaque stability but also induction of phenotypic switching balance out their more pro-atherogenic effects on leukocyte recruitment and lesional foam cell formation. Hence, while considering the therapeutic targeting potential of chemokines in general one should keep in mind that chemokine blocking could reduce plaque size at costs of reduced lesion stability. Still, some chemokines may be useful as biomarkers since augmented levels of CCL5 or CXCL12 correlate with the severity of atherosclerosis or CAD respectively.

Cytokines like IL1-β, IL-6, IL-22, TNFα and GDF-15 seem to particularly foster EC activation and drive early atherogenesis while the effects of IL-33, PGs and LTs are less clear. In addition, IL1-β and IL-22 are also involved in VSMC proliferation and phenotypic switching, but animal models also suggest that IL-1β is beneficial in an advanced stage of the disease by maintaining plaque stability. Caution is also warranted when blocking IL-6 and TNFα. Blocking of those two cytokines enhances total cholesterol burden, LDL, and triglyceride profiles despite improving endothelial function and reducing vascular remodeling. Therefore, to minimize the detrimental effects of elevated lipids, it is important to combine IL-6 and TNFα blocking with lipid lowering strategies like statin treatment. On the other hand, fostering beneficial effects of IL-10 for example to inhibit phenotypic switching of VSMCs could also be a promising therapeutic approach to delay vascular wall remodeling.

Overall, it is important keep in mind that tissue specific expression patterns, the applied model, the prescribed medication and deficiency vs. receptor blocking all differentially impact on well-orchestrated immune functions and, in addition, time and spatial resolution significantly contribute to the results summarized above.

## Author Contributions

BE, AY, and YD performed literature research, drafted the manuscript, and made the figures. IB, SB, EV, and MS wrote the manuscript and provided corrections. All authors have read and agreed to the published version of the manuscript. All authors contributed to the article and approved the submitted version.

## Funding

This research was funded by the Swiss National Foundation (SNF) Project IDs 310030_197655 and 4078P0_198297 to YD and CRSII5_193694 to IB as well as by the Swiss Heart Foundation, Project ID FF20099 to YD, a grant from the Interdisciplinary Center for Clinical Research within the faculty of Medicine at the RWTH Aachen University and NWO-ZonMw Veni (91619053) to EV.

## Conflict of Interest

The authors declare that the research was conducted in the absence of any commercial or financial relationships that could be construed as a potential conflict of interest.

## Publisher's Note

All claims expressed in this article are solely those of the authors and do not necessarily represent those of their affiliated organizations, or those of the publisher, the editors and the reviewers. Any product that may be evaluated in this article, or claim that may be made by its manufacturer, is not guaranteed or endorsed by the publisher.
